# Effects of different nitrogen fertilizers on two wheat cultivars: An integrated approach

**DOI:** 10.1002/pld3.89

**Published:** 2018-10-22

**Authors:** Federico Vita, Beatrice Giuntoli, Simona Arena, Fabrizio Quaranta, Edoardo Bertolini, Valentina Lucarotti, Lorenzo Guglielminetti, Massimo Alessio, Andrea Scaloni, Amedeo Alpi

**Affiliations:** ^1^ LINV‐Department of Plant Soil and Environmental Science University of Florence Florence Italy; ^2^ A.R.E.A. Foundation Pisa Italy; ^3^ Biology Department University of Pisa Pisa Italy; ^4^ Institute of Life Sciences Scuola Superiore Sant'Anna Pisa Italy; ^5^ Proteomics and Mass Spectrometry Laboratory I.S.P.A.A.M. National Research Council Napoli Italy; ^6^ Council for Agricultural Research and Agricultural Economics Analysis Unità di ricerca per la valorizzazione qualitativa dei cereali (CREA‐QCE) Rome Italy; ^7^ Department of Agriculture, Food and Environment (DiSAAA) University of Pisa Pisa Italy; ^8^ Proteome Biochemistry Unit IRCCS‐San Raffaele Scientific Institute Milan Italy; ^9^ Present address: Donald Danforth Plant Science Center Saint Louis Missouri

**Keywords:** 2‐DE, mass spectrometry, nitrogen uptake, qPCR, *Triticum durum*

## Abstract

Investigation of cultivated plant physiology grown under low energy input plays an important role to indicate their fitness to the new environmental conditions. The durum‐wheat cultivars Creso and Dylan were tested to evaluate the growth, production, and proteomic and transcriptomic profiles of the crop under different synthetic and organic nitrogen fertilization regimes. In this work, a two‐dimensional gel electrophoresis (2‐DE) approach combined with liquid chromatography–mass spectrometry (LC–MS) was used to investigate the protein changes induced by the use of different nitrogen sources (hydrolysate of proteins 1 and 2, rhizovit, synthesis, leather) on wheat plants. Proteomic studies were integrated with qPCR analysis of genes related to glutamine synthetase/glutamine‐2‐oxoglutarate aminotransferase (GS‐GOGAT) and tricarboxylic acid (TCA) metabolic pathways because most relevant for nitrogen‐dependent plants growth. The proteomic analysis lead to the isolation of 23 spots that were able to distinguish the analyzed samples. These spots yielded the identification of 60 proteins involved in photosynthesis, glycolysis, and nitrogen metabolism. As an example, the quinone oxidoreductase‐like protein and probable glutathione *S*‐transferase GSTU proteins were identified in two spots that represents the most statistically significant ones in Dylan samples. Transcript analysis indicated that related genes exhibited different expression trends; the heat map also revealed the different behaviors of the hydrolysates of the proteins 1 and 2 nitrogen sources. The effects of nitrogenous fertilizers at the proteomic and agronomic levels revealed that plants fertilized with synthesis or rhizovit gave the best results concerning yield, whereas rhizovit and protein hydrolysates were most effective for proteins content in the grain (% of dry weight). Therefore, all parameters measured in this study indicated that different kinds of nitrogen fertilization used have a relevant impact on plant growth and production.

## 1 INTRODUCTION

The efficient usage of fertilizers by crops is a highly desirable trait both economically and environmentally, despite the traditionally focus of plant breeding on yield (Raun & Johnson, 1999; Vita et al., 2016).

For many decades, cereal production systems have intensified (the so‐called “green revolution”) by resorting to many types of important factors, including the use of large quantities of nitrogen fertilizers, especially in wheat cultivation. However, in the last few decades, an opposite trend has been strengthening as a result of the high cost of fertilizers in plant production and their dispersion in the field, which gives rise to soil and water pollution; moreover, their extensive use is believed to contribute to global warming through emissions of nitrous oxide (Masclaux‐Daubresse et al., 2010). Therefore, it is now important to breed cultivars that can absorb and utilize nitrogen more efficiently to reduce environmental pollution (Bahrman et al., 2004) and meet the needs of modern agriculture, which aims to reduce the input of fertilizers and improve grain quality without affecting yield. This can be achieved by enhancing plant nitrogen economy through the manipulation of nitrogen recycling.

Organic farming methods rely on almost opposite techniques to those used by the green revolution, with the aim of producing healthier and higher quality crops (Guarda, Padovan, & Delogu, 2004). Organic farming is distinguished from conventional agriculture because no chemical pesticides, no synthetic manure, and no genetically modified organisms (GMOs) are permitted (Verhoog, Matze, Van Bueren, & Baars, 2003).

Innovations in production have been evolving toward low‐cost, organic, sustainable, and environmentally friendly systems that must contemporarily ensure the yield and high quality of crops (Calvo, Nelson, & Kloepper, 2014; Du Jardin, 2015). Some authors have proposed the use of biostimulants in plant nutrition to reduce or substitute for the use of inorganic fertilizers, by relying on the positive impact of biostimulants on nutrient and water uptake or utilization (Russo & Berlyn, 1991; Vernieri, Ferrante, Borghesi, & Mugnai, 2006).

Nevertheless, the composition of biostimulants is partly unknown. Furthermore, biostimulants exert a complex action as a consequence of their multiple roles in plants: in fact, they are known to act as promoters of the defense response to biotic and abiotic stress mechanisms, as well as phytonutrients (Bulgari, Cocetta, Trivellini, Vernieri, & Ferrante, 2015; Colla, Rouphael, Canaguier, Svecova, & Cardarelli, 2014; Subbarao, Hussain, & Ganesh, 2015). This complexity can be approached using molecular biology, exploiting tissue‐specific transcriptomic or microarray data to identify target genes related to biostimulants (Santaniello et al., 2012). The transcriptomic approach can be integrated with the analysis of protein profiles, which is an optimal method for quantifying changes in protein abundance caused by cropping systems (Fanucchi et al., 2012; Tétard‐Jones et al., 2013). The main advantage of a proteomic approach lies in the possibility to observe posttranslational changes that would not be identified in the transcriptome. Upon identification of proteins with a changing abundance, candidate genes for agronomic traits can be identified, leading to the development of functional molecular markers that are useful to accelerate and assist crop‐breeding practices (Varshney, Graner, & Sorrells, 2005).

Wheat is one of the three most important cereal crops worldwide. Understanding the uptake, assimilation, and utilization of nitrogen to improve its efficient recovery in grain has been a key goal in cereal research (Shewry, 2009). In well‐aerated soils, the nitrification process dominates, resulting in a low level of ammonium and high production of nitrate, which in turn is the most predominant form of nitrogen used by plants (Crawford & Forde, 2002).

Nitrate uptake occurs at the root level where two nitrate transport systems (NRT1 and NRT2 families) coexist and act coordinately (Masclaux‐Daubresse et al., 2010; Tsay, Chiu, Tsai, Ho, & Hsu, 2007). Further downstream, the first step in nitrate assimilation is its reduction to nitrite, which is catalyzed by nitrate reductase (NR). Nitrite is then translocated to the chloroplast, where it is further reduced to ammonium by nitrite reductase (NIR) (Meyer & Stitt, 2001). In the chloroplast, ammonium assimilation into amino acids is eventually carried out by the so‐called GS/GOGAT cycle (Miflin & Lea, 1980), the major enzymes of which are glutamine synthetase (GS) and glutamate synthase (also known as glutamine‐2‐oxoglutarate aminotransferase [GOGAT]).

Recently, Nigro et al. (2016) have examined the role of GS in terms of nitrogen use efficiency (NUE) and grain protein content in durum wheat: despite some degree of genotypic variation, higher values of GS activity and expression are displayed by genotypes with high grain protein contents, and vice versa.

The relative contribution of the flag leaf to the final nitrogen level in the grain is essential due to its peculiar ability to translocate the assimilates efficiently until the very late stages of leaf senescence (Lopes et al., 2006).

Considering these premises, the objectives of the study presented herein were to compare the effect of organic and conventional fertilizing systems on (a) the wheat flag leaf proteome, (b) plant growth and production, and (c) the transcriptomic profile of the nitrogen uptake pathway. This strategy represents a step toward identifying functional molecular markers for subsequent marker‐assisted breeding of wheat.

## 2 MATERIALS AND METHODS

### 2.1 Experimental design and plant material

**Figure 1:**
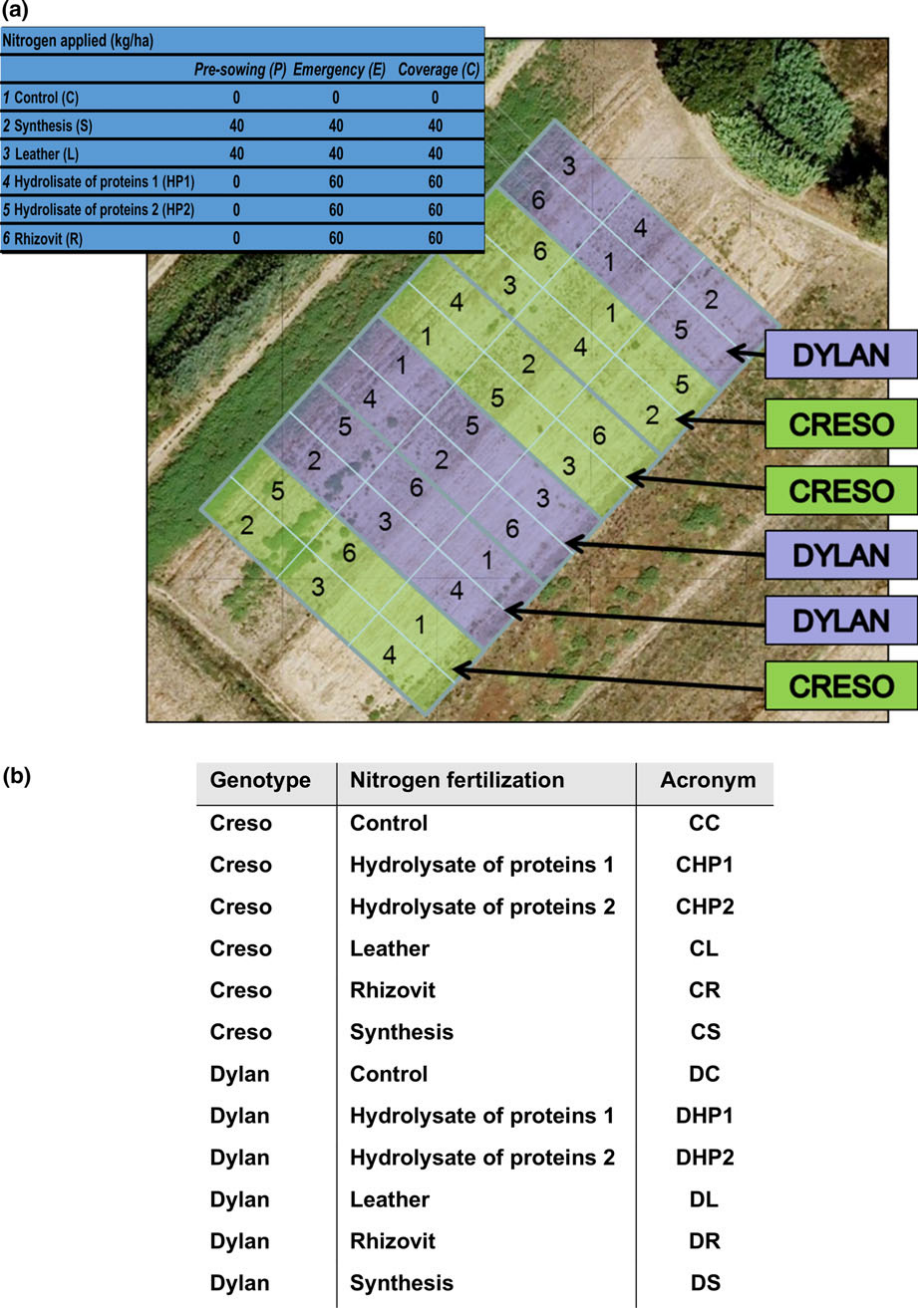
(a) Field experimental design. Numbers indicate different types of nitrogen fertilizations, evaluated in triplicate for each cultivar tested (Creso or Dylan): (1) control, (2) synthesis, (3) leather, (4) hydrolysate of protein 1, (5) hydrolysate of protein 2, and (6) rhizovit. Nitrogen fertilization is reported as kg N ha^−1^. (b) List of samples and relative types of nitrogen fertilization used in this work. CC: Creso control; DC: Dylan control; CHP1: Creso hydrolysate of proteins 1; DHP1: Dylan hydrolysate of proteins 1; CHP2: Creso hydrolysate of proteins 2; DHP2: Dylan hydrolysate of proteins 2; CL: Creso leather; DL: Dylan leather; CR: Creso rhizovit; DR: Dylan rhizovit; CS: Creso synthesis; DS: Dylan synthesis

All experiments were performed on the durum‐wheat (*Triticum durum*) cultivars Creso (registered in 1974) and Dylan (registered in 2002), cultivated at the CRA‐QCE experimental farm in Rome over 2 years, 2011 and 2012. Cultivar pedigrees are available online at http://www.wheatpedigree.net. Plants were treated according to different fertilization regimes that are normally used in organic and conventional agriculture (Figure 1a,b). In detail, six fertilizers were used in the present work. Three of them are commonly used in organic farming, namely Leather meal (indicated as “L” in this study) and the two hydrolysates of protein Ilsadrip Forte N9 (“HP1”) and Protifert N8.3 (“HP2”); the other three fertilizers, urea, ammonium nitrate, and Rhizovit N20 (“R”), derive from chemical synthesis. Urea and ammonium nitrate were used in combination in the treatment named Synthesis (“S”). Figure 1a reports the experimental design and the nitrogen form applied during each treatment. Details about fertilizer composition, soil analysis, and climate data are reported as Supplemental data (Supporting Information Figure S1, Tables S1–3).

Plant growth (represented by plant height and total culm number) and production (cereal yield, hectoliter weight, and 1000‐kernel weight) parameters were measured separately over the 2 years of analysis to evaluate the performance linked to different nitrogen fertilizers and/or different genotypes.

Furthermore, semolina samples were collected after the milling phase (Cyclotec 1093‐Tecator/Hoganas, Sweden) and used for the following analysis: protein content (micro‐Kjeldhal, Nx5.7), sodium dodecyl sulfate (SDS) sedimentation test (3% solution, AACC 56‐70), gluten index (ICC 158, Glutomatic System Perten, Sweden), and rheological parameter (alveographic W, alveograph Chopin, UNI 10453). Semolina color was also measured as the yellow index (Minolta Chromameter CR‐300, CEN standard method 15465).

Plant growth and production data were analyzed using principal component analysis (PCA) and their results were graphically processed to highlight the contribution of each variable class (measured parameters) in the sample differentiation; before analysis, data were subjected to linear transformation by subtracting their respective means and dividing by their squared roots of standard deviations, with the aim to standardize the range of independent variables (the aforementioned parameters).

Statistical analysis was performed on overall data coming from each year of experimentation (2011, 2012) using XLSTAT version 2014.5.03.

Plant material for all analyses was collected at the late developmental stage of the flag leaf, which produces a large proportion (at least 75%) of the photosynthates (carbohydrates) needed for grain filling. Samples (20 g for each biological replicate) were then stored at −80°C before proteomic and transcriptomic analyses.

### 2.2 Proteomic analysis

Leaves (1 g) were ground in liquid nitrogen and homogenized with 1 ml of extraction buffer (5 M urea, 2 M thiourea, 40 mM Tris–HCl, 2% CHAPS, 50 mM DTT). The homogenates were centrifuged for 15 min at 15,000 *g*. Supernatants were precipitated using TCA (15%, v/v) containing 0.007% β‐mercaptoethanol in acetone at −20°C for 2 h and then at 4°C for a minimum of 2 h. Samples were then centrifuged at 4°C for 15 min at 14,000 *g*, supernatants were discarded, and pellets were washed twice with ice cold acetone containing 0.007% β‐mercaptoethanol.

Pellets were dissolved in a rehydration buffer (5 M urea, 2 M thiourea, 4% CHAPS, 40 mM DTT). Protein quantification was performed using a Bradford‐based kit assay (Bio‐Rad Hercules, CA).

Isoelectric focusing (IEF) of total proteins was performed using 18‐cm long immobilized pH gradient (IPG) strips pH 4–7 (GE‐Healthcare). The protein sample was mixed with a rehydration buffer, 0.5% IPG buffer (v/v) of respective pH range, and 0.002% bromophenol blue to a final volume of 340 μL and loaded onto the IPG strips by passive rehydration, 100 or 1000 μg for analytical and preparative gels, respectively. IEF was carried out at 200 V for 3 h, 1000 V for 1 h, 2000 V for 1 h, 3500 V for 1 h, and 56 kV h using the Multiphore II system (Amersham Pharmacia Biotech). IPG strips were then incubated twice in an equilibration buffer [6 M urea, 30% glycerol (v/v), 50 mM Tris–HCl, 2%] SDS for 15 min. The first equilibration was done in the presence of 1.2% DTT (w/v), while in the second incubation, DTT was replaced by 1.5% iodoacetamide (w/v). SDS‐PAGE was performed on 12.5% polyacrylamide gels using a BioRad Protean II XI vertical gel electrophoresis chamber.

Analytical gels for image analysis were stained with silver nitrate as described by Oakley, Kirsch, and Morris (1980), while the preparative gels for the MS analysis were stained with Coomassie brilliant blue (CBB) according to the manufacturer's instructions (Sigma‐Aldrich). Three independent biological replicates, each with three technical replicates (*n* = 9 for each experimental condition), were run for analytical gels while a single technical replicate was run for preparative gel.

Analytical and preparative 2‐DE gel images were acquired at 300 dpi resolution using the ProXpress CCD camera system (Perkin Elmer) and saved as TIF files for image analysis. Spot detection, quantification, and differential expression analysis were performed using Nonlinear Progenesis Same Spots software (Nonlinear Dynamics, Newcastle upon Tyne, UK, version 3.2.3) as previously reported by Vita et al. (2013). Selected protein spots differentially expressed by diverse nitrogen treatments were chosen for further MS analysis on the basis of their ANOVA scores (q‐value) and fold change as estimated by software, with selected spots that showed *a* > 1.2 and *a* > 0.5 fold changes for upregulated and downregulated, respectively, if compared with control sample. Post hoc analysis (Fisher's least significant difference [Fisher's LSD] test) was carried out on ANOVA results, for data coming from each cultivar (Creso and Dylan); CC (Creso control), and DC (Dylan control) were used as reference samples. Protein spot data were also used to perform an agglomerative hierarchical clustering (AHC) analysis, based on dissimilarity through squared Euclidean distance. Heat maps were depicted for both the genotypes using ascendant hierarchical clustering based on Euclidian distances.

Selected spots were manually excised from gels, chopped, and proteins were in‐gel reduced, S‐alkylated and digested with bovine trypsin (Roche Diagnostics Corp.) overnight (Scippa et al., 2010). Digest aliquots were subjected to a desalting/concentration step on a C18 ZipTip microcolumn using 5% formic acid/50% acetonitrile as an eluent before nanoLC‐ESI‐LIT‐MS/MS analysis. Samples were analyzed using a LTQ XL mass spectrometer (Thermo Finnigan, San Jose, CA, USA) equipped with a Proxeon nanospray source connected to an Easy‐nanoLC (Proxeon, Odense, Denmark). Peptide mixtures were separated on an Easy C18 column (10 × 0.075 mm, 3 mm) using a linear gradient from 5% to 50% of acetonitrile in 0.1% formic acid, over 24 min, at a flow rate of 300 nl/min. Spectra were acquired the range *m*/*z* 400–2000. Acquisition was controlled by a data‐dependent product ion scanning procedure over the three most abundant ions, enabling dynamic exclusion (repeat count 1 and exclusion duration 1 min). The mass isolation window and collision energy were set to *m/z* 3 and 35%, respectively.

MASCOT software package (Matrix Science, UK) was used to identify protein spots unambiguously from an updated wheat non‐redundant sequence database from UniprotKB (taxonomy, Viridiplantae) by using a mass tolerance value of 2.2 Da for a precursor ion and 0.8 Da for MS/MS fragments, trypsin as a proteolytic enzyme, a missed cleavages maximum value of 2, and Cys carbamidomethylation and Met oxidation as fixed and variable modifications, respectively. Candidates with more than two assigned peptides with the MASCOT score >25 (*p* ≤ 0.01 for a significant identification) were further evaluated by the comparison of their calculated mass value with that obtained from SDS‐PAGE. Where appropriate, protein identification was checked manually to provide for a false positive rate less than 1%. Identified proteins were reported according to their Exponentially Modified Protein Abundance Index (emPAI), with a cutoff value of 0.5.

Proteins obtained without functional identification were then used for Protein Blast Analysis (UniProtKb blast *p*) performed with default settings.

### 2.3 Total RNA extraction, primer design, and real‐time PCR analysis

Total RNA was extracted from pulverized samples as described in Chomczynski and Sacchi (2006). RNA integrity was evaluated by agarose gel electrophoresis using 1% agarose gel, followed by spectrophotometric quantification, and quality control as described in (Fleige & Pfaffl, 2006). A quantity of 1 μg of total RNA was processed with the Maxima First Strand cDNA Synthesis Kit (Thermo Fisher Scientific) in a reaction volume of 10 μl for removal of contaminating DNA and RNA reverse transcription. Gene expression analysis was carried out using an ABI Prism 7300 sequence detection system (Applied Biosystems, USA) as described by (Licausi et al., 2010). Quantitative PCR was performed using 20 ng cDNA and iQ™ Sybr Green Supermix (BioRad laboratories), according to the manufacturer's instructions. Two technical replicates were performed for each biological replicate (*n* = 3). To analyze the expression of genes related to the Krebs cycle and nitrogen assimilation metabolism, 21 qPCR primers pairs were designed (Supporting Information Table S4) using the tool Primer 3 (http://primer3.ut.ee/), applying different strategies. Sixteen primer pairs were designed starting from transcript sequences obtained from Krasileva et al. (2013) using the *Arabidopsis* gene identifiers as a query to retrieve *T. durum* orthologs. Among those, polyubiquitin 10 was used as housekeeping gene.

Four primer pairs (see Supporting Information S4) were designed after multi‐alignment of sequences belonging to monocot species closely related to wheat, namely *Oryza sativa* var. *japonica*,* Brachypodium distachyon*,* Setaria italica*, and *Sorghum bicolor*. Such sequences were recovered through blast search (Altschul, Gish, Miller, Myers, & Lipman, 1990), providing individual *Arabidopsis thaliana* sequences as the query in each case.

One primer pair was designed on available *Triticum aestivum* sequence (Bernard et al., 2008).

Relative gene expression levels were calculated with the 2^−ΔΔCt^ method (Livak & Schmittgen, 2001) and plotted into heat maps using ascendant hierarchical clustering based on Euclidean distances. Statistical analysis was performed using XLSTAT version 2014.5.03. Furthermore, relative expression data for three selected genes (*GLU, CSY,* and *NRT*) were subjected to one‐way ANOVA analysis, followed by Tukey's honestly significant difference (Tukey's‐HSD) post hoc test (*p* ≤ 0.05). To examine patterns of genetic variation, further two‐way ANOVA analysis was also carried out with the gene (different isoforms or transporter) and fertilization treatment as main factors.

## 3 RESULTS

### 3.1 Results of experimental field trials

Field experiments were carried out over two subsequent years, and crop production was recorded; the major climatic difference between the 2 years is represented by the level of rainfall that caused different levels of production and hence high standard deviations. Nevertheless, the productivity results (Table 1) clearly showed that the Dylan cultivar performed better in terms of cereal yield, hectoliter weight, and total culms; this trend was confirmed at the technological level, as shown, in particular, by the yellow and gluten indexes. In contrast, the Creso cultivar showed high values for 1000‐kernel weight and protein content. Based on the data concerning both the total nitrogen level and the microbiological activities (Biolog EcoPlate™) measured in the experimental fields, it can be deduced that all the experimental sites were substantially uniform (Supporting Information Table S2).

**Table 1:**
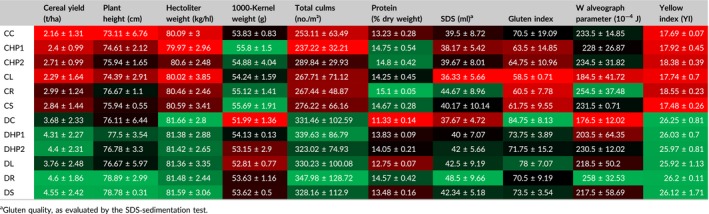
Results related to experimental field trials performed over 2 years. Data are reported as the mean ± standard deviation. Measured parameters were divided based on their role: plant growth and kernel production parameters are indicated in bold and technological parameters in italic. Mean values ± standard deviations for each parameter are individually represented by a colorimetric scale: minimum (red) and maximum (green) value through black. Further information about measured parameters is reported in the Material and Methods section

**Figure 2:**
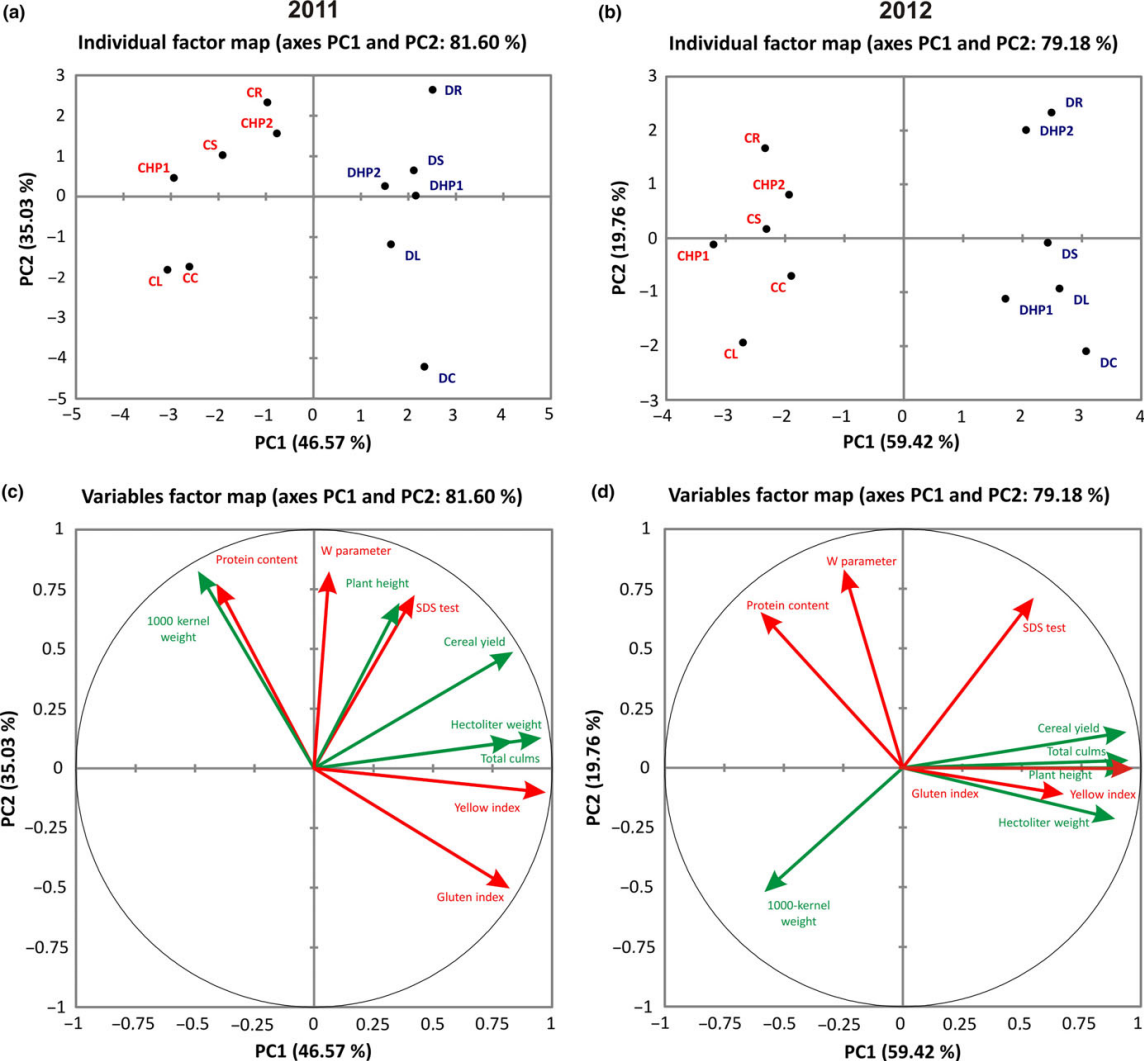
Principal component analysis of field trial results over the 2 years of experimentation. (a, b) Individual factor map linked to the distribution of sample according to the multifactorial analyses results. PC1, first dimension; PC2, second dimension. (c, d) Variable factor map related to the contribution of each class of crop parameters. The length of the vectors is directly correlated to their significance, while the angle α formed between two vectors, or between a vector and an axis, provides an indication of a positive correlation for 0 ≤ α < 90° (*r* close to 1), negative correlation for 90° < α ≤ 180° (*r* close to −1), and no linear dependence for α = 90° (*r* close to 0)

Additionally, values reported in Table 1 as means of 2 years were examined separately by year by PCA (Figure 2). The PCA analyses were conducted separately on the results obtained for the 2 years of experimentation to determine whether, despite the high agronomical and technological differences (Table 1), the relationships between the samples were substantially constant. For either year of experimentation, the samples clustered according to the genotype (axis 1, PC1), as well as on the base of the different types of nitrogen fertilization (axis 2, PC2) (Figures 2a,b). These results were confirmed by the high quantity of total variance collected by PCA analysis in both years (81.60%, 2011; 79.18%, 2012).

A variable factor map was generated to highlight the correlation among variables (Figures 2c,d). The results related to the first year (2011, Figure 2c) showed a general positive correlation among variables (measured parameters), with the exception of the comparison between the gluten index and 1000‐kernel weight/protein content, for which there was a negative correlation. In the second year of analysis (2012, Figure 2d), many variables mainly displayed a positive correlation both among them and with axis 1 (PC1); however, a negative correlation was instead reported between the SDS‐sedimentation parameter and 1000‐kernel weight, as well as protein and the W alveographic index, against many variables, such as hectoliter weight and gluten index. These trends suggest that the relationships among the measured parameters may change depending on the year.

### 3.2 Two‐dimensional gel electrophoresis (2‐DE) analysis and protein identification

**Figure 3:**
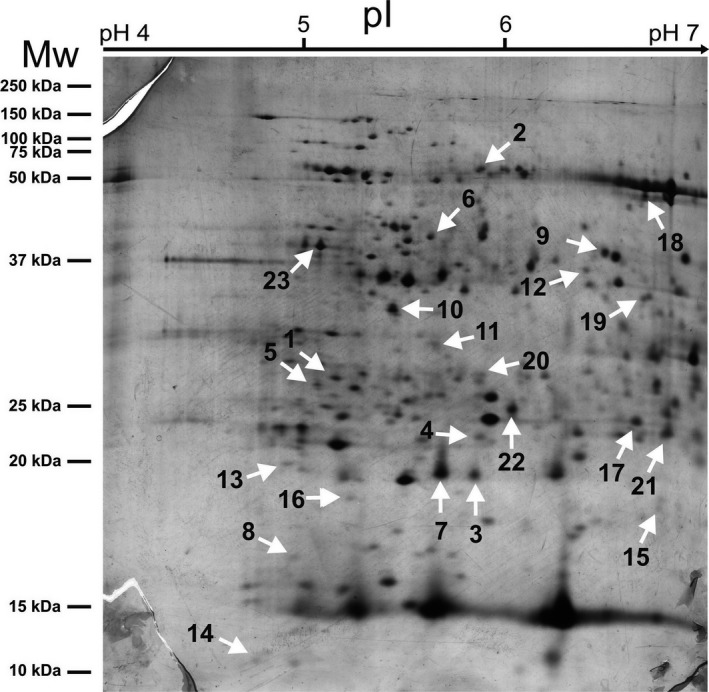
Representative 2D gel from a Dylan synthesis sample (0.1 mg of protein extract) subjected to the silver staining procedure. White arrows indicate spots selected by bioinformatics analysis

**Figure 4:**
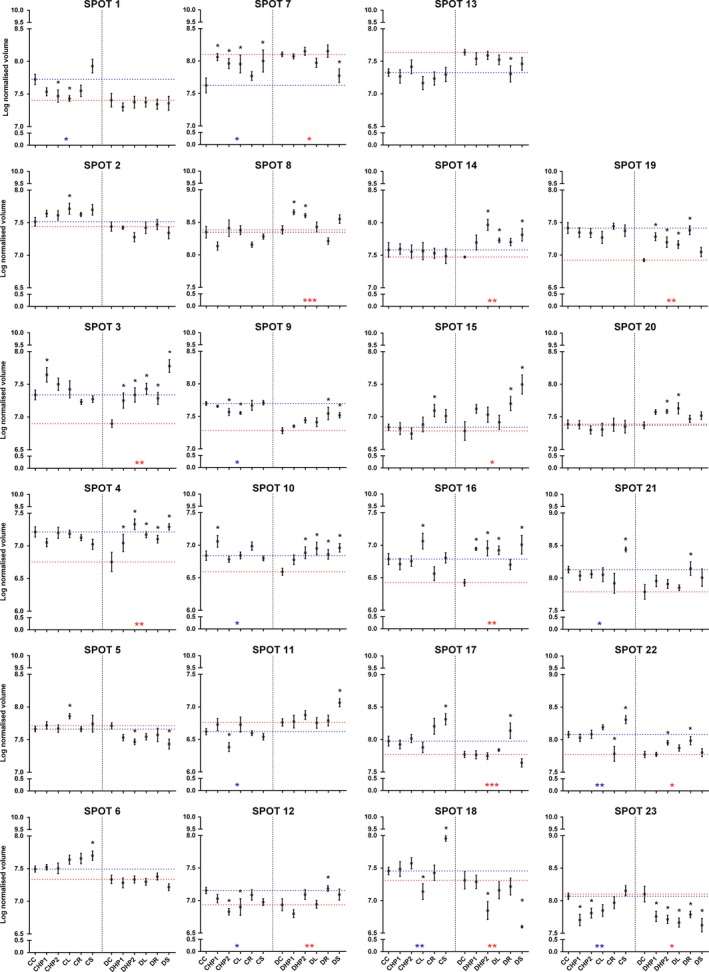
Normalized intensity levels of the spots selected for MS analysis. The relative amount of signal for each spot is expressed as the log10 normalized volume (spot optical density). Values are means ± SEM (*n* = 9). For each spot, the value corresponding to the control samples was projected on the *y*‐axis and represented as dotted lines (CC: blue line; DC: red line), to ease comparison between control and treated samples within each cultivar. Statistical significance was evaluated by one‐way ANOVA (blue, Creso, and red, Dylan, asterisks) followed by Fisher LSD test (see Table 2 for a summary of the test) performed through pairwise comparisons with the reference sample (CC: Creso control; DC: Dylan control). Black asterisks mark statistically significant treatments. Data are reported with *p*‐values. *p ≤ 0.05; **p ≤ 0.01; ***p ≤ 0.001; ****p ≤ 0.0001. Samples are indicated by acronyms, as specified in Figure 1b

Flag leaf samples were collected (Figure 1b) and used for proteomic analyses. Analytical gels (Figure 3) allowed us to detect more than 900 reproducible protein spots. After bioinformatics analysis, 23 candidate spots were selected based on both their statistical significance and fold change as able to discriminate the effect of different nitrogen fertilization regimes in the analyzed genotypes. Intensity values recorded for these selected spots (Figure 4) were analyzed separately for each cultivar. One‐way ANOVA and post hoc (Fisher's LSD) test were used to evaluate the overall significance within each genotype to highlight the presence of significant trends due to different types of nitrogen fertilization (Figure 4 and Table 2). Most of the spots (1, 2, 3, 4, 6, 9, 10, 12, 14, 15, 16, 17, 18, 19, 21, and 22) displayed higher intensity in Creso than in Dylan under control conditions, while spots 5, 7, 8, 11, 13 and 23 showed the opposite situation and spot 20 had the same intensity in both cultivars (Figure 4). Most of the treatments produced significant results in only one cultivar, while only in a few cases was significance observed in both cultivars: for instance, protein hydrolysate 1 (HP1) significantly affected the intensity of spot 3 and 23 in both Dylan and Creso. This genotype‐specific behavior supports the suitability of the selected spots for cultivar discrimination.

**Table 2:** Results of Fisher's least significant difference (LSD) test on relative spot intensity, calculated by comparing the various nitrogen treatments with the respective untreated control (CC and DC)

Spot	CHP1	CHP2	CL	CR	CS	DHP1	DHP2	DL	DR	DS
1		*	*							
2			*							
3	*					*	*	**	*	****
4						*	****	**	**	***
5			*				*			*
6					*					
7	**	*	*		*					*
8						*	*			
9		*	*						*	*
10	*						*	**	*	*
11		*								*
12		**	*						*	
13									*	
14							***	*		*
15				*					*	**
16			*			**	***	**		***
17					*				***	
18			*		**		*			**
19						**	*	*	****	
20							*	**		
21					*				*	
22				**	*		*		*	
23	**	*				*	**	**	*	**

Data are reported as *p*‐values. *p ≤ 0.05; **p ≤ 0.01; ***p ≤ 0.001; ****p ≤ 0.0001.

Based on the one‐way ANOVA analysis, the most significant spots for the different nitrogen treatments were 8 and 17 in Dylan and 18, 22, and 23 in Creso (Table 2). The one‐way ANOVA results for spot 23 (Figure 4) were statistically significant for both cultivars; the post hoc test indicated that Creso samples treated with protein hydrolysates (CHP1, CHP2), as well as all Dylan samples, had significantly lower protein expression. Different trends are reported for spot 7 and 18, for which data related to fertilizer of synthesis (S) were significant in both cultivars but upregulated for Creso and downregulated for Dylan; spots 15 and 22 were, in relation to rhizovit (R) treatment, statistically significant in Creso and Dylan but upregulated in Dylan, while only spot 15 was upregulated in Creso. Spot 16 was significant in the samples treated with leather (L).

All candidate spots were subjected to mass spectrometry analysis, which led to the identification of 60 proteins (Table 3). Proteins from each spot were sorted according to enrichment values (emPAI) for relative quantification, with a cutoff value of 0.5. To allow functional identification, protein blast (default settings) was performed when no other information was available (Supporting Information Table S5).

**Table 3:** Proteins identified by ESI‐Quad TOF analysis. Proteins were sorted based on their emPAI score (cutoff value = 0.5). Shading denotes uncharacterized (light gray) and predicted (dark gray)

Spot no.^a^	Protein list	Acc. No.^b^	Organism	Protein name	Score^c^	Seq. Cov. %^d^	Mw Kda Obs/Theo^e^		pI Obs/Theo^f^		Length__Prot_	Red./Unique peptides^g^		emPAI^h^
1	1	I1IB68	*Brachypodium distachyon*	Elongation factor Tu	412	18	27.2	50.5	5.17	5.88	471	11	6	2.39
	2	Q10KF0	*Oryza sativa* subsp. *japonica*	Proteasome subunit alpha type‐2	484	38	27.2	25.8	5.17	5.36	235	8	8	1.08
	3	O04905	*Arabidopsis thaliana*	UMP‐CMP kinase	179	11	27.2	22.5	5.17	5.79	417	4	4	0.6
2	4	M8BWQ5	*Aegilops tauschii*	Alanine aminotransferase 2	116	6	57.5	57.9	5.88	5.62	523	3	3	0.48
	5	C7IXC7	*Oryza sativa* subsp. *japonica*	Os01 g0791033 protein	154	18	57.5	28.8	5.88	6.35	262	4	4	0.45
	6	Q42971	*Oryza sativa* subsp. *japonica*	Enolase	131	6	57.5	48.0	5.88	5.41	446	2	2	0.36
3	7	P12810	*Triticum aestivum*	16.9‐kDa class I heat shock protein 1	321	41	19.5	16.9	5.86	5.83	151	5	4	0.65
4	8	I3T490	*Lotus japonicus*	Uncharacterized protein	322	37	22.3	20.9	5.89	5.77	189	9	7	2.78
	9	P12810	*Triticum aestivum*	16.9‐kDa class I heat shock protein 1	543	60	22.3	16.9	5.89	5.83	151	12	9	2.1
	10	F2EH52	*Hordeum vulgare* var. *distichum*	Predicted protein	409	53	22.3	17.5	5.89	5.54	158	10	8	1.93
5	11	I1H026	*Brachypodium distachyon*	Uncharacterized protein	1114	74	26.3	25.7	5.07	8.6	247	30	16	10.69
	12	Q7XCK6	*Oryza sativa* subsp. *japonica*	Chitinase 8	400	32	26.3	24.8	5.07	5.65	261	5	5	1
	13	I1IB68	*Brachypodium distachyon*	Elongation factor Tu	252	13	26.3	50.5	5.07	5.88	471	4	4	0.63
6	14	Q40073	*Hordeum vulgare*	Ribulose bisphosphate carboxylase/oxygenase activase A	1948	59	41	46.1	5.63	5.6	464	120	32	15.11
	15	I1GXN4	*Brachypodium distachyon*	Uncharacterized protein	220	13	41	45.6	5.63	6.05	425	4	4	0.65
	16	F2D751	*Hordeum vulgare* var. *distichum*	Predicted protein	232	15	41	40.3	5.63	6.04	374	4	4	0.56
7	17	Q40073	*Hordeum vulgare*	Ribulose bisphosphate carboxylase/oxygenase activase A	837	42	19.6	46.1	5.67	5.6	464	18	15	1
	18	M0YB06	*Hordeum vulgare* var. *distichum*	4‐Hydroxy‐4‐methyl‐2‐oxoglutarate aldolase	121	9	19.6	23.8	5.67	5.8	219	2	2	0.5
8	19	I1I6Q9	*Brachypodium distachyon*	Uncharacterized protein	185	13	16.8	39.8	4.92	8.29	384	4	3	0.96
	20	O03042	*Arabidopsis thaliana*	Ribulose bisphosphate carboxylase large chain	325	14	16.8	52.7	4.92	5.88	479	14	7	0.56
9	21	F2CR16	*Hordeum vulgare* var. *distichum*	Fructose‐bisphosphate aldolase	634	31	38.9	37.9	6.51	6.06	360	18	9	2.94
	22	Q40073	*Hordeum vulgare*	Ribulose bisphosphate carboxylase/oxygenase activase A	735	32	38.9	46.1	6.51	5.6	464	18	13	1.12
	23	K3Y823	*Setaria italica*	Uncharacterized protein	239	13	38.9	41.1	6.51	6.24	376	4	4	0.68
	24	M0WP40	*Hordeum vulgare* var. *distichum*	Uncharacterized protein	118	9	38.9	23.2	6.51	5.6	217	3	3	0.6
	25	F2CXT7	*Hordeum vulgare* var. *distichum*	Fructose‐bisphosphate aldolase	243	13	38.9	38.9	6.51	6.39	358	7	5	0.53
	26	Q0J8A4	*Oryza sativa* subsp. *japonica*	Glyceraldehyde‐3‐phosphate dehydrogenase 1	205	15	38.9	36.3	6.51	6.67	337	4	4	0.52
	27	C6TBN2	*Glycine max*	Probable aldo‐keto reductase 1	167	11	38.9	38.3	6.51	6.14	346	3	3	0.51
10	28	A2YUR3	*Oryza sativa* subsp. *indica*	Putative uncharacterized protein	240	15	32.9	32.0	5.30	5.88	309	3	3	0.49
	29	P52428	*Oryza sativa* subsp. *japonica*	Proteasome subunit alpha type‐1	145	15	32.9	29.6	5.30	5.37	270	3	3	0.32
	30	F2E3Y2	*Hordeum vulgare* var. *distichum*	Predicted protein	149	9	32.9	65.5	5.30	5.04	593	4	4	0.3
	31	P38076	*Triticum aestivum*	Cysteine synthase	102	8	32.9	34.1	5.30	5.46	325	2	2	0.3
	32	P12810	*Triticum aestivum*	16.9‐kDa class I heat shock protein 1	175	23	32.9	16.9	5.30	5.83	151	2	2	0.29
	33	C5YSP7	*Sorghum bicolor*	Fructose‐bisphosphate aldolase	84	5	32.9	41.9	5.30	6.39	389	2	2	0.16
11	34	Q9THX6	*Solanum lycopersicum*	Thylakoid lumenal 29 kDa protein	571	27	30.1	29.3	5.65	7.91	345	16	10	3.31
12	35	F2CQP0	*Hordeum vulgare* var. *distichum*	Predicted protein	521	27	35.9	37.4	6.41	8.25	347	10	7	2.42
	36	Q9ZP06	*Arabidopsis thaliana*	Malate dehydrogenase 1	392	18	35.9	33.3	6.41	6	341	13	6	2.13
	37	M0UNQ9	*Hordeum vulgare* var. *distichum*	Uncharacterized protein	175	9	35.9	48.3	6.41	8.93	464	10	3	0.84
	38	F2W3J6	*Rosa roxburghii*	Rubisco activase	313	28	35.9	29.5	6.41	5.85	265	6	6	0.76
	39	Q76MX2	*Solanum tuberosum*	Bifunctional L‐3‐cyanoalanine synthase/cysteine synthase 1	202	14	35.9	36.9	6.41	5.79	351	5	4	0.66
	40	M0Y8P6	*Hordeum vulgare* var. *distichum*	Uncharacterized protein	168	16	35.9	25.4	6.41	5.56	240	4	3	0.54
13	41	O03042	*Arabidopsis thaliana*	Ribulose bisphosphate carboxylase large chain	612	17	19.9	52.7	4.91	5.88	479	33	10	9.1
	42	M8BDP7	*Aegilops tauschii*	Uncharacterized protein	442	35	19.9	25.6	4.91	8.58	236	21	7	3.28
	43	P26667	*Triticum aestivum*	Ribulose bisphosphate carboxylase small chain PW9	539	62	19.9	14.9	4.91	5.85	175	13	11	1.18
14	44	P26667	*Triticum aestivum*	Ribulose bisphosphate carboxylase small chain PW9	383	57	11.2	14.9	4.74	5.85	175	16	9	0.8
15	45	C7IXC7	*Oryza sativa* subsp. *japonica*	Os01 g0791033 protein	228	21	18.6	28.8	6.76	6.35	262	5	5	1.2
	46	P00871	*Triticum aestivum*	Ribulose bisphosphate carboxylase small chain PWS4.3	149	34	18.6	14.8	6.76	5.85	174	5	5	0.5
16	47	I6MHV0	*Hordeum vulgare* var. *distichum*	Ribulose bisphosphate carboxylase large chain	126	30	18.7	27.7	5.23	5.74	249	17	8	1.24
17	48	Q10CE7	*Oryza sativa* subsp. *japonica*	Probable glutathione *S*‐transferase GSTU1	339	23	23.8	25.8	6.66	5.9	231	10	5	1.69
	49	O03042	*Arabidopsis thaliana*	Ribulose bisphosphate carboxylase large chain	344	18	23.8	52.7	6.66	5.88	479	8	8	0.56
	50	D4N8D8	*Secale cereale x Triticum durum*	Carbonic anhydrase	264	24	23.8	28.1	6.66	8.35	259	4	4	0.53
18	51	K4A9B8	*Setaria italica*	Uncharacterized protein	569	26	46.9	51.6	6.71	6.65	477	14	12	2.88
	52	I3T490	*Lotus japonicus*	Uncharacterized protein	199	24	46.9	20.9	6.71	5.77	189	4	4	0.94
19	53	O03042	*Arabidopsis thaliana*	Ribulose bisphosphate carboxylase large chain	1239	41	33.6	52.7	6.70	5.88	479	65	23	3.9
	54	Q69LA6	*Oryza sativa* subsp. *japonica*	Probable pyridoxal biosynthesis protein PDX1.1	221	15	33.6	33.7	6.70	6.4	318	4	4	0.64
20	55	P46226	*Secale cereale*	Triosephosphate isomerase	924	62	27.3	26.8	5.88	5.24	253	37	15	7.43
	56	I1HWV6	*Brachypodium distachyon*	Uncharacterized protein	392	38	27.3	27.3	5.88	5.67	242	9	8	1.62
21	57	P28524	*Hordeum vulgare*	Superoxide dismutase [Mn] (fragment)	666	77	22.7	2.2	6.8	3.49	20	19	12	3.65
22	58	Q00434	*Triticum aestivum*	Oxygen‐evolving enhancer protein 2	629	43	25.0	20.0	6.04	5.95	258	29	11	7.05
23	59	P26302	*Triticum aestivum*	Phosphoribulokinase	192	9	29.2	39.2	5.07	4.99	404	3	3	0.6
	60	Q0J0H4	*Oryza sativa* subsp. *japonica*	Pyruvate dehydrogenase E1 component subunit beta‐2	290	13	29.2	36.6	5.07	4.95	376	4	4	0.5

Spot number corresponds to those reported in Figure 2.

UniProtKB accession number.

Mascot score.

Percent sequence coverage.

Gel‐observed vs. theoretical molecular weight.

Observed vs. theoretical isoelectric point; *observed and theoretical weight/pi may differ on the basis of the real molecular weight of the protein in *Triticum durum*.

Number of matched redundant and unique peptides.

Exponentially modified protein abundance index (emPAI) referring to the relative quantification of protein.

### 3.3 Agglomerative hierarchical clustering and heat map analysis of spot intensities

**Figure 5:**
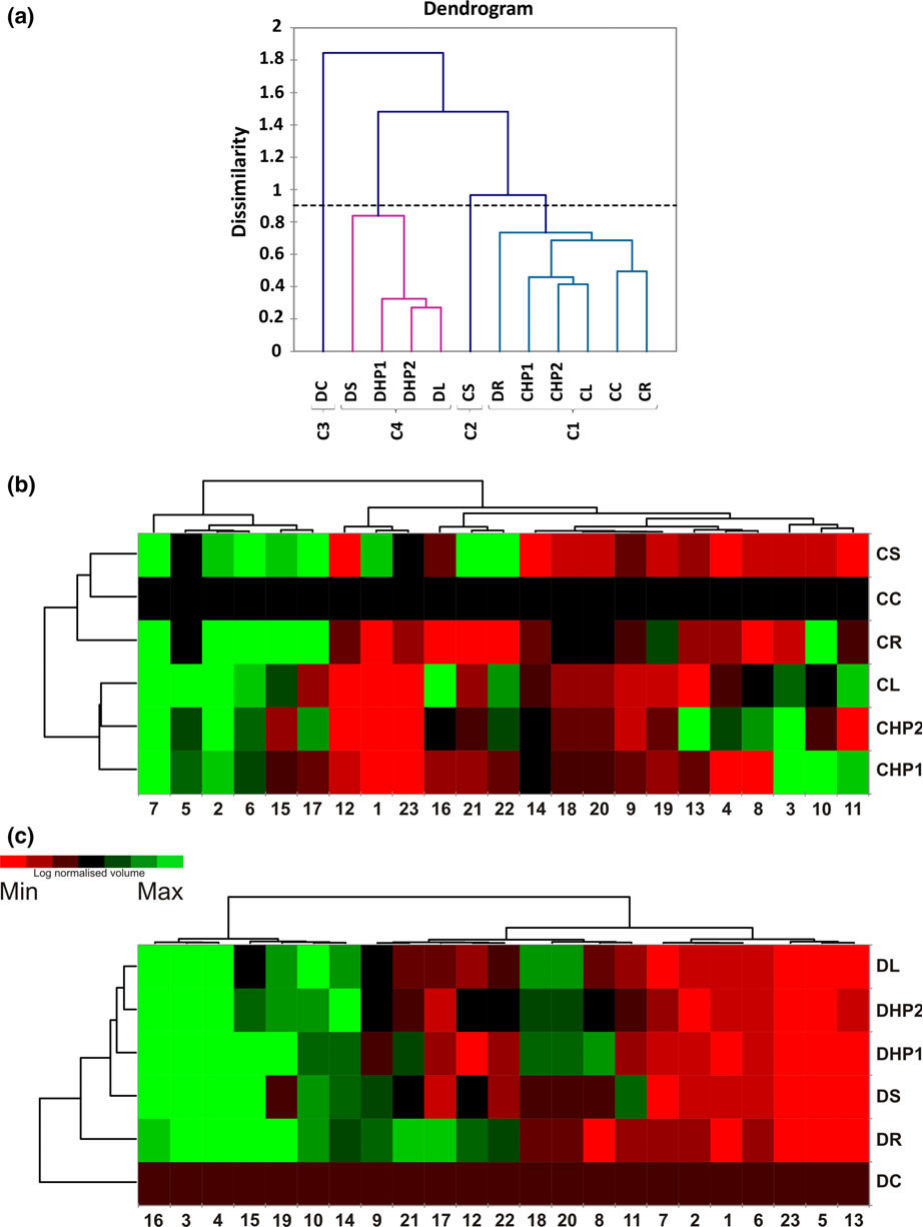
Clustering analysis of the quantitative protein spot data. (a) Dendrogram output of the agglomerative hierarchical clustering (AHC) analysis of spot intensity. (b) Heat map representation of the spot intensity data in the Creso cultivar. The CC sample was used as an internal standard (log normalized volume = 1). (c) Heat map representation of the spot intensity data in the Dylan cultivar. The DC sample was used as an internal standard (log normalized volume = 1). Sample names in rows are indicated by acronyms specified in Figure 1b; spot numbers, in columns, correspond to those indicated in Figure 4

The quantitative behavior of the 23 selected discriminative spots was examined by AHC to group samples based on the global differences in spot intensity. The generated dendrogram distinguished the samples in four clusters (clusters C1–C4, Figure 5a). Clusters C3 and C2 were composed of a single sample (DC and CS, respectively), while C4 consisted of 4 Dylan samples and C1 grouped all Creso samples and the remaining sample of Dylan (DR). These data allowed us to assert that samples were mainly grouped on a genotypic basis. Moreover, common trends could be observed in the distribution of certain treatments: in particular, HP1, HP2 ,and L showed the same effects in both cultivars. This result indicated that the use of organic fertilizers has, from a proteomic perspective, a similar effect in the analyzed two cultivars. An analogs observation can also be applied to synthetic fertilizers, mainly urea (S), which greatly affects both the Dylan and Creso cultivars (Figure 5a).

Quantitative data obtained by gel analysis of the 23 spots (i.e., their log10‐normalized volumes) were also displayed as two heat maps (one for each cultivar) and clustered by means of Euclidean distances (Creso, Figure 5b, and Dylan, Figure 5c). In this way, two dendrograms were generated, one related to samples (clustering by rows) and the other to the selected spots (clustering by columns). The results of the Creso cultivar (Figure 5b) showed that the first dendrogram (left side) collected samples in two clusters, the first being the CS, CC, and CR samples, while the second consisted of the CL, CHP2, and CHP1 samples. The results of the second dendrogram (top side) allowed division of the spots into three main clusters based on differences that can be attributed to the Creso cultivar samples. Starting from the left of the image, the first cluster included 6 spots (2, 5, 6, 7, 15, and 17), which were upregulated with respect to CC. Spots included in the aforementioned cluster also appeared to be upregulated in two samples, R and S, which are both synthetic fertilizers. The second cluster included three spots (1, 12, and 23) with opposite trends, demonstrating a downregulation with respect to the control sample; the only exception was, in spot 1, the CS sample. Spots grouped in this cluster showed that samples with leather (L) and protein hydrolysates (HP1, HP2) were downregulated when compared to synthetic fertilizer samples (R, S). The third cluster, however, had a less homogeneous distribution in which the spots were divided into four subclusters. In the first subcluster, three spots (16, 21, and 22) were grouped with generally heterogeneous expression levels, such as CS (upregulated) and CR (downregulated) samples. The second subcluster included five spots (9, 14, 18, 19, and 20) that were downregulated with respect to the control, with the exception only of the CR sample (spot 19). The third subgroup also included three spots (4, 8, and 13), in which the samples exhibited lower intensity levels than the controls, with the exception of the CHP2 sample, which showed an upregulation with respect to the control. Finally, the fourth and last subcluster showed a mixed pattern in which the intensity of the three remaining spots (3, 10, and 11) had heterogeneous values, with extremes represented by the CS (downregulated) and CHP1 (upregulated) samples.

The heat map related to the Dylan cultivar (Figure 5c) showed a different distribution of the data: the first dendrogram (left side) grouped four main clusters; the first three clusters included one sample each of DC, DR, and DS. The fourth cluster included the samples DL, DHP1, and DHP2, grouping them in the same way as previously observed for the Creso cultivar.

The second dendrogram (Figure 5c, top side) shows the spots grouped into three main clusters based on their quantitative trends. Starting from the left side of the image, the first cluster grouped seven upregulated spots (3, 4, 10, 14, 15, 16, and 19), whereas the second cluster grouped nine spots (8, 9, 11, 12, 17, 18, 20, 21, and 22) with intermediate values; spots 18 and 20 were upregulated in three samples (DL, DHP1,and DHP2) and downregulated in the other treated samples (DS, DR), whereas spot 9 showed an opposite trend. The third cluster finally grouped seven downregulated spots (1, 2, 5, 6, 7, 13, and 23) compared with the control sample (DC), two of which (1 and 23) were mostly also downregulated in the Creso cultivar. However, considering the various clusters, the overall picture emerges from the absence of apparent differences between treatments in Dylan despite the positive results obtained in the Creso cultivar.

### 3.4 qPCR analysis of the selected genes

**Figure 6:**
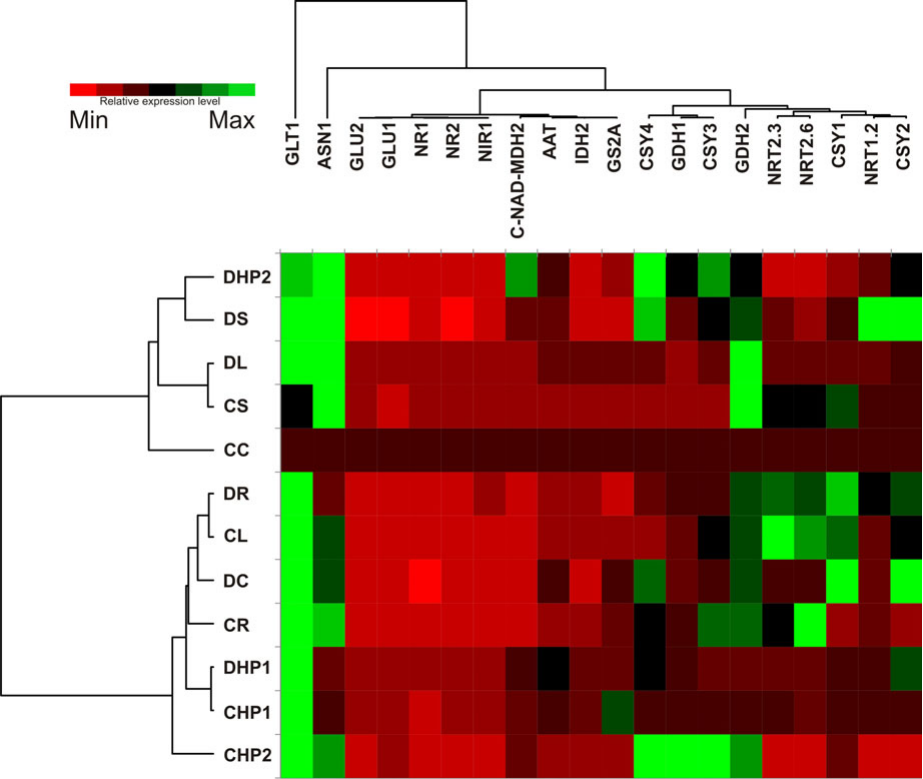
Heat map of the relative gene expression of selected candidate markers for nitrogen fertilization in wheat. Data are the mean values of gene expression (*n* = 3). The expression in the reference sample CC was set as 1 for each gene. Sample names and gene identities are specified in Figure 1b and Supporting Information Table S4, respectively. MDH2 = Cytosolic‐NAD‐dependent malate dehydrogenase 2 (*C‐NAD‐MDH2*). Reference data for the gene expression analysis can be found in Supporting Information Table S6

The previously described assessment provided suitable protein‐based molecular markers for the discrimination of flag leaf responses to the distinct fertilization systems of interest in the two cultivars under consideration. In an attempt to understand whether transcript‐based markers could be validly employed for the same purpose, samples were subjected to a targeted qPCR analysis generated with primers built on *T. durum* sequences. Selected genes belonging to the GS‐GOGAT (glutamine synthetase‐glutamate synthase) and tricarboxylic acid (TCA) metabolic pathways were evaluated, as they are relevant for nitrogen uptake and use and, thus, likely to respond to different fertilization treatments. Further information regarding the qPCR results is provided in Supplemental Material (Supporting Information Table S6). In the heat map, the samples (Figure 6, left side diagram) allowed the data to be separated into two clusters. Starting from the top of the picture, the first cluster grouped five samples, two related to Creso (CC and CS) and three related to the Dylan sample (DS, DHP2, and DL), with a different degree of differentiation from that reported by the dendrogram (Figure 6, left side). The second cluster displayed a most complex scenario, where samples like CR and CHP2 exhibited specific behaviors while some others were grouped based on their treatment (CHP1 and DHP1); this latter observation highlighted a common response reported in both cultivars.

Hierarchical clustering of gene expression patterns shed light on the behavior of the genes across samples. Four main clusters of gene expression were detected (Figure 6, top side diagram). Two of them resulted from only single gene, *GLT1* (a NADH‐dependent glutamate synthase) and *ASN1* (a glutamine‐dependent asparagine synthetase), which displayed elevated expression levels across most of the other samples when compared to CC. *GLT1* encodes one of the GOGAT isoforms (NADH‐GOGAT) present in plants (Temple, Vance, & Gantt, 1998). Nigro et al. (2013) have reported the presence of two homologous genes (NADH‐GOGAT‐3A and NADH‐GOGAT‐3B) in durum wheat that exhibit different expression levels in leaves collected during different developmental stages, with a slight decrease observed during the grain filling phase.

An increase in NADH‐GOGAT expression has also been reported for plants during leaf senescence (Gregersen & Holm, 2007; Kichey, Le Gouis, Sangwan, Hirel, & Dubois, 2005). Glutamine‐dependent asparagine synthetase 1 (*ASN1*) catalyzes the formation of asparagine in an ATP‐dependent reaction that utilizes glutamine as a nitrogen source. Avila‐Ospina, Marmagne, Talbotec, Krupinska, and Masclaux‐Daubresse (2015) have reported that asparagine synthetase (AS) is needed more in senescing leaves when barley (*Hordeum vulgare* L.) plants are grown under high nitrate than when they are grown under nitrate‐limiting conditions. In our experiments, the relative expression levels reported for this gene displayed a different trend depending on the analyzed samples; these results may be explained by taking into consideration how the different nitrogen sources may have concomitantly released different nitrogen quantities.

Our results obtained using leaves close to the senescence stage generally showed high expression levels in all the treatments, with the exception of the CC and CS samples.

A third expression cluster grouped nine genes together: *GLU2* (glutamate synthase 2), *GLU1* (glutamate synthase 1), *NR1* (nitrate reductase 1), *NR2* (nitrate reductase 2), *NIR1* (ferredoxin nitrite reductase), *C‐NAD‐MDH2* (cytosolic‐NAD‐dependent malate dehydrogenase 2), *AAT* (aspartate aminotransferase), *IDH2* (isocitrate dehydrogenase [NAD] subunit 2), and *GS2a* (GS2 plastid glutamine synthetase isoform). In *Arabidopsis*, two coding genes for Fd‐GOGAT, *GLU1* and *GLU2,* encode enzymes that are localized in plastids, in which the GLU1 exhibits the highest expression in leaves while *GLU2* is mostly expressed in roots (Coschigano, Melo‐Oliveira, Lim, & Coruzzi, 1998; Kissen et al., 2010; Temple et al., 1998). These genes generally exhibited the lowest expression levels across the analyzed samples, with the only remarkable exception of *C‐NAD‐MDH2*, which was highly expressed in DHP2. This enzyme was considered for qPCR analysis by virtue of its key role in the generation of 2‐oxoglutarate for ammonium assimilation and amino acid biosynthesis (Fernie, Carrari, & Sweetlove, 2004); interestingly, in a previous study, the same enzyme has been found to be highly expressed in *Zea mays* leaves treated with protein hydrolysate (Schiavon, Ertani, & Nardi, 2008).

The last cluster of transcripts included those genes that varied the most among the samples. This cluster may be further divided into four branches. The first branch includes *GDH1* (glutamate dehydrogenase 1) and two isoforms of citrate synthase, *CSY3* and *CSY4*. The two remaining citrate synthase isoforms (*CSY1*,* CSY2*) were placed in the fourth branch together with the low‐affinity nitrate transporter *NRT1.2*. GDH protein is a hexamer comprised of two subunit polypeptides (α and β) that differ slightly in mass and charge (Purnell, Skopelitis, Roubelakis‐Angelakis, & Botella, 2005). Since the GOGAT cycle is the major route of ammonium assimilation in plants, GDH may participate in primary and secondary ammonium assimilation, playing a complementary role to the GOGAT cycle. Approximately 95% of ammonia that is available to plants is assimilated via the GS/GOGAT pathway. As previously stated, it is now clear that the GDH enzyme plays a negligible role in the assimilation of ammonium (Tercé‐Laforgue et al., 2013). Figure 6 shows the different responses of these two genes to the treatments (e.g., hydrolysate of proteins).

Samples treated with protein hydrolysates usually showed low expression levels of all citrate synthase isoforms. Indeed, Schiavon et al. (2008) have reported high expression levels of citrate synthase in *Z. mays* plants treated with a specific kind of protein hydrolysate (alfalfa protein hydrolysate). These differences may be linked to many variables, such as the composition of the fertilizer or the plant age. The second branch of the last cluster includes only the glutamate dehydrogenase 2 (*GDH2*). The data showed that the transcripts related to this gene were highly expressed in most samples, with the exception of those treated with the hydrolysate of protein 1 (CHP1, DHP1). Several lines of evidence indicate now that the GDH enzyme plays a negligible role in the assimilation of ammonium (Tercé‐Laforgue et al., 2013). For the most part, nitrate transporters were included in the third branch of the dendrogram, where two of them, the high‐affinity transporters *NRT2.3* and *NRT2.6*, were placed because of their comparable expression trends. In *A. thaliana*, NRT2.3 and 2.6 proteins displayed high homology values in protein sequence, but NRT2.3 exhibited a nitrate‐inducible pattern in shoot tissues (Okamoto, Vidmar, & Glass, 2003). In contrast, NRT2.6 exhibited an expression pattern that was induced by high nitrogen levels.

Considering the qPCR trends to differentiate samples, specific relationships may be observed between specific genes and some of the fertilization regimes. Control samples (CC and DC), in which no fertilization was used, exhibited a similar low expression level of most of the reported genes (Supporting Information Table S6), with the exception of NADH‐dependent glutamate synthase 1 (*GLT1*) and two citrate synthase isoforms (*CSY1* and *CSY2*) with higher expression levels in the Dylan sample. Samples treated with protein hydrolysate 1 (CHP1, DHP1) displayed several genes with common quantitative trends in Creso and Dylan, as observed by the heat map sample dendrogram (Figure 6, left side).

Samples fertilized with protein hydrolysate 2 (CHP2, DHP2) showed that the citrate synthase isoform (*CSY4*) and asparagine synthetase (*ASN1*) exhibited the same quantitative trends in samples belonging to both cultivars; the latter gene was also constitutively expressed in samples fertilized with urea and ammonium nitrate (CS, DS).

With regard to the leather treatment, samples treated with such fertilizer (CL, DL) displayed comparable expression levels of the glutamate dehydrogenase 2 (*GDH2*) gene.

**Figure 7:**
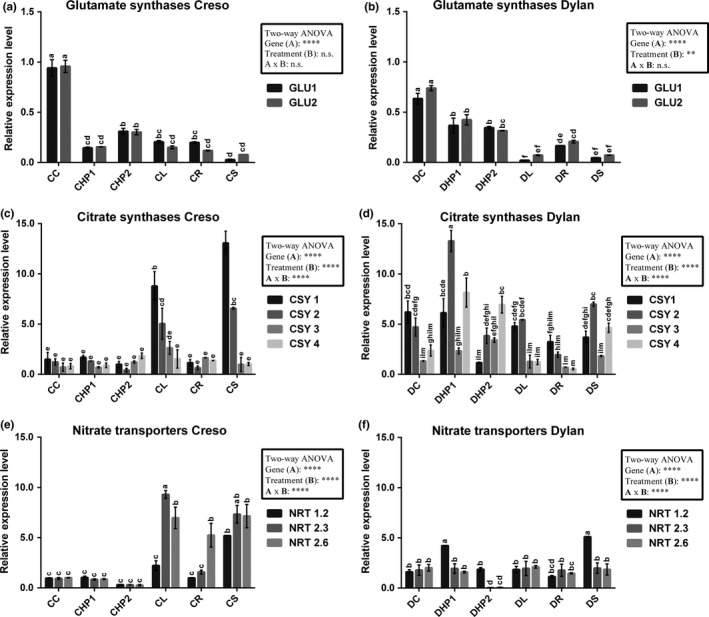
Graphs related to the relative expression levels reported for the selected isoforms. Representation of qPCR results related to glutamate synthase (a, b), citrate synthases (c, d), and nitrate transporters (e, f) for both the cultivars. Data are reported as the mean of the relative expression levels ± standard deviations (*n* = 3), using CC as the reference sample for each isoform. Letters refer to the output of one‐way ANOVA followed by Tukey's post hoc test. Two‐way ANOVA results are, instead, summarized in the insets. CSY: citrate synthase; GLU: glutamate synthase; NRT: nitrate transporter. Samples are indicated by acronyms, as specified in Figure 1b

To investigate specific changes in GLU (glutamate synthase), CSY (citrate synthase) isoforms, and nitrate transporters (NRT), data were plotted separately in histograms (Figure 7). These three activities of nitrate transporters, citrate synthase, and glutamate synthase were selected because they play important roles in the transport and assimilation of nitrate in plants (Foyer, Noctor, & Hodges, 2011).

The GLU results showed that control samples of Creso (CC, Figure 7a) and Dylan (DC, Figure 7b) had the highest expression values, while surprisingly, synthesis treatment (CS, DS) exerted the lowest effect. Two‐way ANOVA results for Creso cultivar showed a high significance level only for the gene factor (different GLU genes, *p* ≤ 0.0001), whereas statistical significance was found for the Dylan for gene (*p* ≤ 0.0001) as well as treatment factors (*p* ≤ 0.01).

Citrate synthase is considered one of the most important enzymes in the TCA cycle because it catalyzes the reaction that controls the rate of the respiratory pathway (Douce & Day, 2012).

Conversely, as stated previously, nitrogen assimilation through the GS/GOGAT pathway is fundamental for the cell, but assimilation is closely related to respiration because GS and GOGAT require ATP and carbon skeletons: the early steps in the TCA cycle represent the source for such skeletons, and therefore, citrate synthase plays a pivotal role in the carbon/nitrogen interaction (Nunes‐Nesi, Fernie, & Stitt, 2010). The results related to CSY indicated that citrate synthase isoforms usually exhibited only two Creso samples, CL and CS, with high expression levels (Figure 7c), whereas most of the Dylan samples (Figure 7d) generally displayed higher expression levels for most of the samples, as confirmed by the one‐way ANOVA results; the relative expression level of these genes should take into account that the expression of control Dylan (DC) was higher than that reported for the Creso control (CC). Overall, the results may be linked to the different growth parameters previously reported (Table 1, Figure 2), for which the Dylan cultivar has been recorded as having the best productive performance in most fertilization regimes. Schiavon et al. (2008) have reported the expression levels of some genes, such as citrate synthase 1, which are not influenced in *Z. mays* plants supplied with protein hydrolysate as a fertilizer; in our case, no effect was reported on CSY1 in samples fertilized with the hydrolysate of proteins (HP1, HP2).

Two‐way ANOVA results showed, for both the cultivars, significant effects of gene (*p* ≤ 0.0001) and treatment (*p* ≤ 0.0001), as well as their interactions (*p* ≤ 0.0001).

Nitrate transporter data showed that two Creso samples (CL, CS, Figure 7e) had the highest expression levels among the analyzed genes. More precisely, the highest expression level of NRT1.2 was recorded in the CS sample, while the NRT2.3 gene was highly expressed in the CL sample.

DHP1 and DS samples showed the highest expression levels for this cultivar (Figure 7f); the NRT1.2 gene was mainly responsible for this variation since it showed 4 times greater expression compared with the control sample (DC).

Two‐way ANOVA results showed, as previously reported for the citrate synthase results, significant effects of the Creso as well as Dylan samples, considering both gene (*p* ≤ 0.0001) and treatment (*p* ≤ 0.0001) factors, as well as their interactions (*p* ≤ 0.0001).

**Figure 8:**
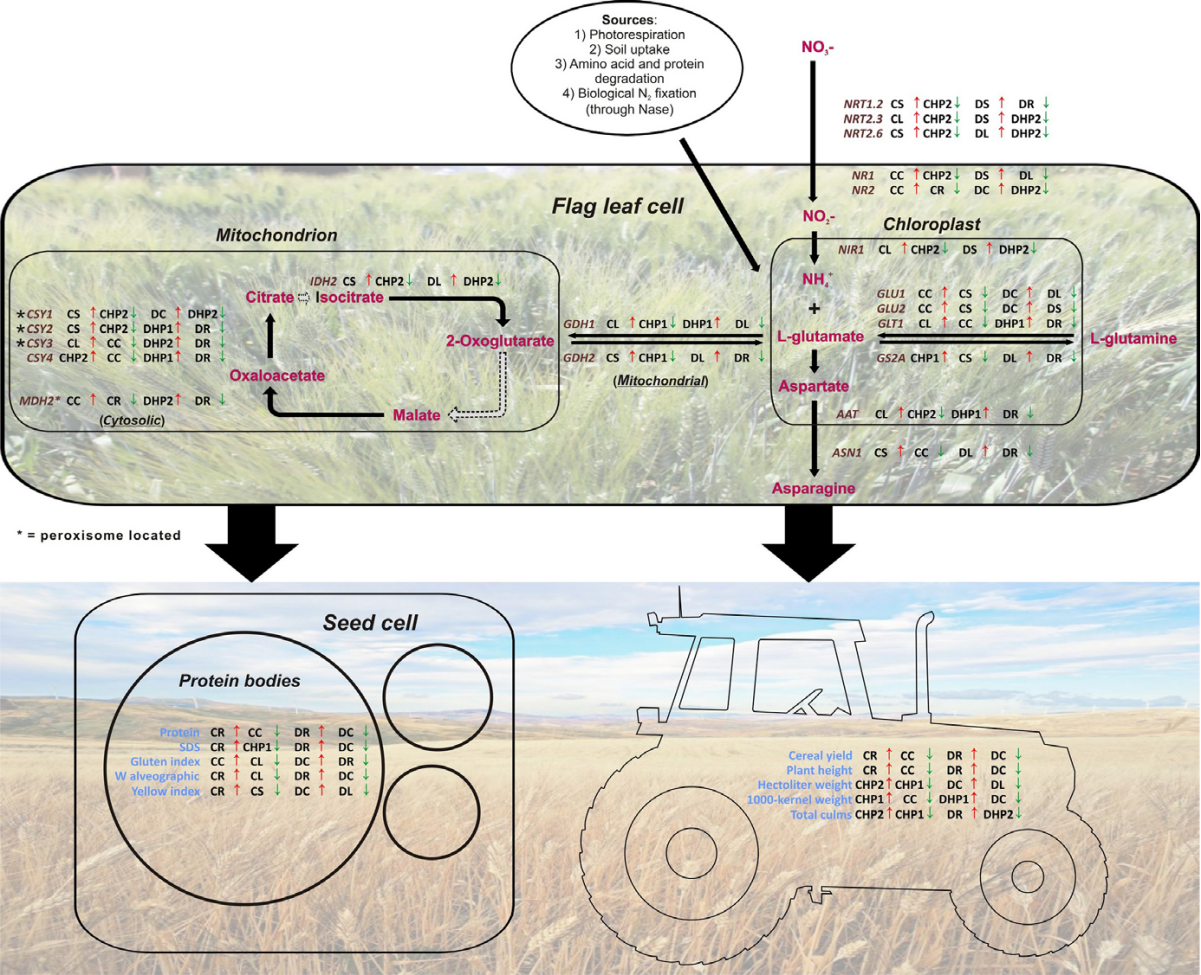
Schematic representation of data related to qPCR (top diagram) and plant growth/production (bottom diagram). Data are reported as minimum and maximum values for each cultivar for the analyzed gene (marked in brown) and parameters (marked in blue). Samples are indicated by acronyms, as specified in Figure 1b. The tractor imagine silhouette was downloaded from http://www.supercoloring.com/it/silhouettes/trattore

The observed effects of fertilizer treatments on agronomic performance and marker gene expression are schematized in Figure 8. The transcriptomic results had the specific trends reported above; in this figure, it appears that plant growth and production parameters confirmed a positive role of rhizovit treatment, while leather produces an opposite effect.

## 4 DISCUSSION

### 4.1 Protein identification and their metabolic role

The overall aim of this research was to investigate the differential effect of conventional and organic nitrogen fertilization systems on the wheat flag leaf proteome. The production results (Table 1 and Figure 2) clearly showed the predominant effect of genotype, although some of the fertilizers, such as rhizovit (R) or leather (L), showed consistent differences in yield rates. The results also revealed a significant difference in the proteome of different cultivars (Figures 4, 5), which might be explained by different growth behaviors and might result in a dissimilar effect of the utilized fertilizers.

Some of the identified proteins could be grouped according to their biological function. Proteins with the highest emPAI value reported in spots 4, 6, 7, 8, 13, 14, 15, 16, and 19 are involved in photosynthetic metabolism (Table 3). Among these proteins, ribulose‐1,5‐bisphosphate carboxylase/oxygenase (RuBisCO) is the major enzyme assimilating CO_2_ into the biosphere. The RuBisCO large subunit was identified with the highest emPAI scores in three different spots (13, 16, and 19; Table 3), whereas the small subunit was identified in spot 14, as well as in spot 15; considering data related to spot intensity, we may observe that many of these spots displayed statistically significant results only for the Dylan cultivar. The expression level of this enzyme has been measured in wheat leaves, showing a continuous increase that was directly proportional to the increase in nitrogen fertilizers (Chandna & Ahmad, 2015). Although the effects of nitrogen on growth and photosynthesis have long been known (Cramer & Lewis, 1993), only recent interest has shown the impact of nitrogen fertilization on photosynthetic physiology. The relationship between photosynthetic electron transport and electron utilization in photosystem II in plants treated with different forms of nitrogen has been investigated in some detail (Mintāle & Vikmane, 2015; Zhou et al., 2011).

Other identified enzymes are involved in the glycolytic pathway, namely fructose‐bisphosphate aldolase (n. 21, spot 9) and triose phosphate isomerases (n. 55, spot 20). Both enzymes have been shown to be differentially expressed in wheat leaves based on nitrogen treatments of the nitrogen starvation‐sensitive variety UP2382, but not the low‐N stress‐tolerant VL616 (Chandna & Ahmad, 2015). According to our results, when the spot intensities of the treated samples were compared to the control, the data linked to aldolase (spot 9) and triose phosphate isomerases (spot 20) showed a generally equal or lower intensity value for Creso samples, whereas the data related to Dylan samples appeared to be higher. The results linked to spot 20 instead indicated that some Dylan results (DHP2 and DL) were significant.

Nitrogen metabolism was also found affected by our experimental conditions. Alanine aminotransferase 2 (n. 4, spot 2) catalyzes the reversible transfer of an amino group from alanine (Ala) to 2‐oxoglutarate to form pyruvate and glutamate (D'mello, 2015). Kendziorek, Paszkowski, and Zagdańska (2012) analyzed the four alanine aminotransferase (AlaAT) homologs in *T. aestivum*, two of which encode AlaAT enzymes, whereas the other two homologs have glutamate (glyoxylate aminotransferase, GGAT) activity. The results showed that GGAT activity was slightly influenced by nitrogen availability that, conversely, regulates AlaAT expression. The results for spot 2 indicated that CL (Creso leather) was the only statistically significant treatment, despite a general quantitative trend showing higher spot intensities in Creso compared with Dylan samples.

Phosphoribulokinase (n. 59, spot 23), an enzyme that catalyzes the ATP‐dependent phosphorylation of ribulose‐5‐phosphate to ribulose‐1,5‐phosphate, may play a role in the carbon and nitrogen balance, as suggested by recent results obtained using *Rhodobacter sphaeroides* (Farmer & Tabita, 2015). Spot 23 also displayed a significant result for data linked to protein hydrolysate 2 (HP2). Aspartate‐semialdehyde dehydrogenase (n. 15, spot 6) and mitochondrial ornithine aminotransferase (n. 51, spot 18) were identified after blast analysis. Aspartate‐semialdehyde dehydrogenase is the second enzyme in the aspartate pathway, whereas ornithine aminotransferase mitochondrial is an essential enzyme that plays a key role in arginine catabolism and hence in nitrogen recycling. Mitochondrial ornithine aminotransferase participates in the catabolic branch of proline metabolism, allowing the recovery of nitrogen that is stored or transported as arginine (Funck, Stadelhofer, & Koch, 2008). The aforementioned spots (6, 18) showed a similar quantitative trend, with the Creso samples usually demonstrating higher intensity values in comparison to Dylan; despite this general trend, spot 6 showed a statistically significant result only for the CS sample, whereas spot 18 provided more statistically significant data for both cultivars (Figure 4).

Elongation factor Tu (or ER1a) identified in spot 1 (n. 1) is a protein that promotes the GTP‐dependent binding of aminoacyl‐tRNA to the A‐site of ribosomes during protein biosynthesis; its protein level significantly changes in response to post‐anthesis fertilization (Altenbach et al., 2011). The quantitative results for spot 1 showed that only Creso samples displayed statistically significant results for CHP2 and CL, which showed lower intensity values when compared with the control sample (CC).

The 20‐kDa chloroplastic chaperonin identified after blast analysis (Supporting Information Table S5) in spot 5 (n. 11) has recently been shown to increase under low nitrogen conditions in *Z. mays* genotypes (Nazir et al., 2016). Data linked to this spot showed significant results in Creso (CL) as well as Dylan samples (DHP2, DS), with an opposite trend compared with the control; the CL sample showed an upregulation, whereas the DHP2 and DS showed a downregulation.

Thylakoid lumenal 29 kDa protein (n. 34, spot 11) belongs to a large group of lumenal proteins with a function that remains mostly unknown; Staudinger et al. (2012) have reported that the expression of this protein is affected in *Medicago troncatula* plants subjected to salt stress. Our results demonstrated that two samples, CHP2 for Creso and DS for Dylan, showed statistically significant differences that were linked to spot intensities. Although oxygen‐evolving enhancer protein 2 (n. 58, spot 22) was downregulated in hydroponically grown 15‐day‐old *Z. mays* plants after nitrate supplementation (Prinsi, Negri, Pesaresi, Cocucci, & Espen, 2009), our results showed that the detected variations were significant for both cultivars in the specific treatments (CR, CS, DHP2, and DR).

Probable glutathione *S*‐transferase (n. 48, spot 17) presumably functions to protect the cell from oxidative damage via the addition of GSH to reactive molecules (Mcgonigle, Keeler, Lau, Koeppe, & O'keefe, 2000). Our results showed that the spot trend (spot 17, Figure 4) displayed significant results only for Dylan samples, which were mainly related to DR samples (Figure 4 and Table 2), similarly to the results reported for some wheat cultivars grown under different nitrogen levels (Chandna & Ahmad, 2015).

Pyridoxal biosynthesis PDX1.1 (n. 54, spot 19) and ornithine oxo‐acid aminotransferase (n. 51, spot 18, Table 3, Supporting Information Table S5) are enzymes that are also involved in the nitrogen metabolism pathway. A recent publication by Khan et al. (2015) has shown that the *PDX1.1* gene is transcriptionally downregulated under conditions of N starvation. Quantitative results related to cultivar Creso in spot 18 were statistically significant in the case of CL and CS, whereas no significant data were reported for spot 19. A different trend was observed for the cultivar Dylan, which showed statistically significant results both for spot 18 (DHP2, DS) and 19 (DHP1, DHP2, DL, DR).

### 4.2 Gene expression modulated by different nitrogen sources

qPCR analysis allowed us to assess the different behavior of the 20 selected genes, related to particular pathways (the GS‐GOGAT metabolism and the TCA cycle) expected to be widely responsive to different forms of available nitrogen. We estimated that this approach would have been more informative than the validation of genes encoding those proteins found as differentially expressed, based on the consideration that most of them belonged to unrelated pathways. Transcriptomics has been used previously to directly identify genes involved in N metabolism and storage protein synthesis, which are differentially expressed in response to organic and conventional fertilizers (Lu et al., 2005). Our results related to the GS‐GOGAT and the TCA cycle confirmed that the different nitrogen fertilizers might induce different flag leaf responses, which was more evident for plants fertilized with one of the protein hydrolysates (HP1, Figure 6).

*GLT1 (*NADH‐GOGAT) and *ASN1* were upregulated in response to most treatments, making them unsuitable for discrimination (Figure 6). Furthermore, *NR* and *NIR* (participating in the nitrate reduction pathway) are known to be induced when nitrogen is present in the form of nitrate, but not in the presence of other N sources such as NH_4_+ (Criado, Roberts, Echeverria, & Barneix, 2007). Our results were in agreement with this trend, showing no evident upregulation in response to the sample treatment.

Glutamate synthase (commonly termed GOGAT) was also considered. In higher plants, it occurs in two distinct isoforms, NADH‐GOGAT and ferredoxin‐dependent GOGAT (Fd‐GOGAT) that differ in many aspects, such as molecular mass, subunit composition, enzyme kinetics, antigenic and reductant specificity, and metabolic function (Temple et al., 1998). No significant upregulation of the two enzymes was measured, although a downregulation could be observed for cultivar Creso (control vs. treated samples) (Figure 6). Kissen et al. (2010) obtained knockdown mutants for the expression of one of the two genes in *A. thaliana* encoding Fd‐GOGAT. In these plants, photosynthesis was sensibly downregulated, while genes related to the plant response to different abiotic stresses (light, drought, salt, heat, and others) were activated.

Cultivar‐specific expression trends were, instead, recorded for citrate synthase (Figure 7). The differences may be linked to two main factors, namely the high level of expression reported in the DC and the different production performances of the two cultivars, as reported in Table 1.

Also nitrate transporter genes, finally, showed partially different regulation among cultivars (Figure 7), that could be linked to the form of nitrogen (nitric or ammonia) preferentially absorbed by each of them. A recent work regarding the cultivar Svevo (Curci et al., 2017) showed that high‐affinity transporters, in particular NRT2.5, are upregulated in roots of nitrogen‐starved plants, although unchanged in leaves. Svevo has been recently established and has comparable characteristics to Creso. Despite these differences between Creso and Dylan, upregulation of one transporter (*NRT1.2*) was reported in both cultivars for samples treated with synthesis fertilizers (S), making it a possible marker of fertilization type; this result may be explained in terms of the fertilizer composition, which consists of a mixture of urea and ammonium nitrate (Supporting Information Table S1).

The GS/GOGAT pathway is known to have a fundamental function in primary nitrogen assimilation, but it also plays a central role in the re‐assimilation of ammonium released by photorespiration; plants can perceive any accumulated ammonium as toxic by activating, consequently, a stress response. The effects of the modified expression level of Fd‐GOGAT are not limited to nitrogen metabolism, but include photosynthesis and, to a minor extent, flavonoid biosynthesis (Kissen et al., 2010). Differences reported in qPCR data may have been mainly due to different types of fertilization input.

The relevance of the glutamate molecule in wheat plants has been demonstrated by our results, but further studies are needed to define glutamate homeostasis in such plants. The role of glutamate as a signaling molecule is well known in the animal kingdom; therefore, a similar role in plants has been hypothesized but not conclusively shown (Forde & Lea, 2007).

Globally, the qPCR results showed trends that were inconsistent with the growth and production data, while the results of the proteomic analysis were more consistent with those data. The chemical properties of fertilizers strongly influence the development of the plant as it can be shown both agronomically and molecularly.

The recent paper published by Curci et al. (2017) represents a comprehensive transcriptomic analysis of durum wheat (cv Svevo) under nitrogen deficiency showing that genes are differentially expressed depending upon the tissue. In our experimentation, many of the differentially expressed genes belong to the carbon metabolism (glycolysis, TCAs) as some orthologs genes of the malate dehydrogenase (MDH, identified in the spot 12) and of the succinate dehydrogenase (SDH) upregulated in the root. Among the other differentially expressed genes, we can identify glutathione *S*‐transferases (GSTs), two isoforms of which are saturated identified as upregulated in the leaves.

The overall results thus show that the evident phenotypic changes are accompanied by a metabolic remodeling, with numerous genes, belonging to diversified metabolic pathways, being induced by reduced nitrogen level. These data could be useful to improve the efficiency of nitrogen use for durum wheat growing.

The modulation in the metabolic response highlighted by the proteomic and transcriptomic analyzes show that samples differed more according to the cultivar they belong to and, to a lesser extent, depending on the applied treatment (i.e., type of fertilizer used). If we consider the production data, synthetic fertilizers (urea, ammonium nitrate, rhizovit) have given the best production performances. In the same way, proteomic data show how the treatments with synthetic fertilizers generically induce an upregulation in the two cultivars considered. By way of example, the data reported in spots 15 and 17 show the most significant differences. The probable glutathione *S*‐transferase GSTU1 protein, identified in spot 17, is an ideal candidate, as the expression of this protein class is influenced, both in the leaves and in the root, under conditions of nitrogen deficiency. Regarding the transcriptomic data, the results obtained are strongly influenced by the type of cultivar considered. As for the Creso cultivar, the CS (Creso synthesis) sample has numerous upregulated genes, followed by the CL (Creso leather) sample. The overall trend concerning the Dylan cultivar is different, where a high number of induced genes is reported in the samples treated with protein hydrolysates (DHP1, DHP2) and, to a lesser extent, in the sample treated with leather (DL). From the physiological point of view, these results could be explained by specific characteristics related to the two cultivars, and in particular to the form of preferentially absorbed nitrogen (nitric or ammoniacal). In fact, the fertilizers used, except synthetic products (ammonium nitrate and rhizovit, Supporting Information Table S1) do not contain any forms of ammonia nitrogen but contain nitrogen exclusively in the nitric form.

In conclusion, the results reported in the present work show how the use of a multidisciplinary approach (agronomic and molecular) has allowed identifying differences due to the different type of nitrogen fertilization; this information could represent a step forward useful in programs of genetic improvement and crop management.

## CONFLICT OF INTEREST

The authors declare that the research was conducted in the absence of any commercial or financial relationships that could be construed as a potential conflict of interest.

## AUTHOR CONTRIBUTIONS

F.V. and A.A. designed the research. F.V., B.G., S.A., V.L., and M.A. performed the experiments and analyzed the data. F.V. performed the statistical analysis. E.B. performed the sequence similarity search to identify homolog sequences. F.Q. handled the experimental field trials and leaf sampling. F.V., E.B., M.A., A.S., and A.A. organized and drafted the paper. L.G., M.A., B.G., and E.B. helped revise the manuscript with all authors contributing to the discussion of the data and to the writing.
