# Supplementary material for: Effects of different nitrogen fertilizers on two wheat cultivars: An integrated approach
Source: Plant Direct. 2018 Oct 22;2(10):e00089. doi: 10.1002/pld3.89 (PMC6508776; doi:10.1002/pld3.89)
Supplement: Supplementary file 1 [file PLD3-2-e00089-s001.docx]

## *Supplemental Material*

**Effects of different nitrogen fertilizers on two wheat cultivars: a proteomic approach**

**Federico Vita, Beatrice Giuntoli, Simona Arena, Fabrizio Quaranta, Edoardo Bertolini, Valentina Lucarotti, Lorenzo Guglielminetti, Massimo Alessio, Andrea Scaloni, Amedeo Alpi***

*** Correspondence:** Corresponding author: amedeo.alpi@gmail.com

**
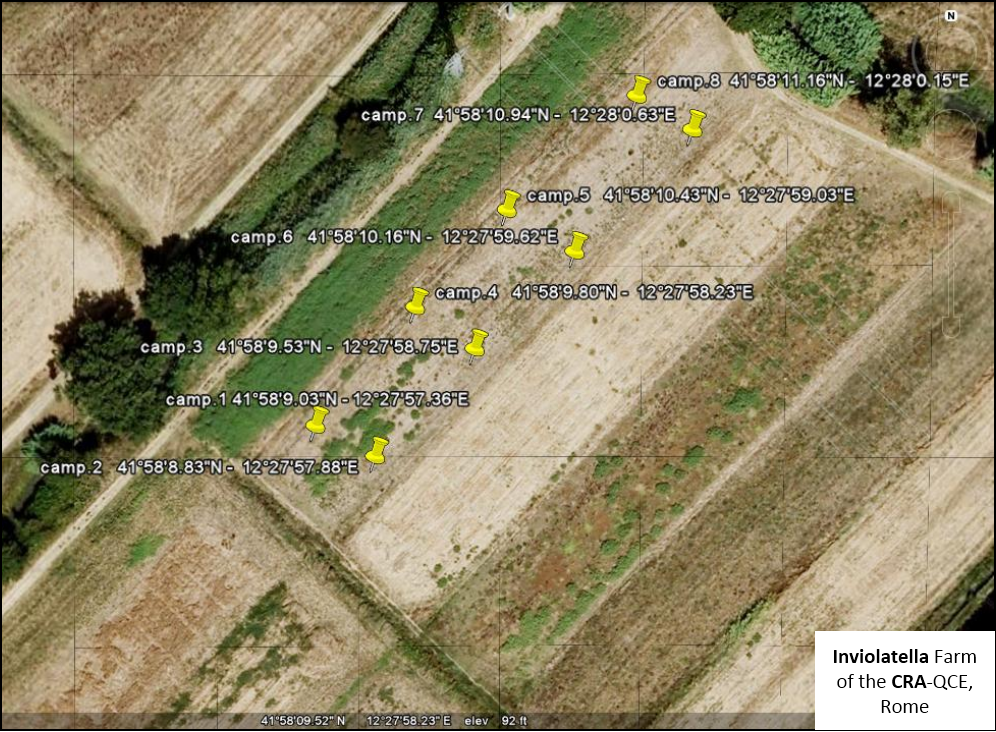
**

**SUPPLEMENTAL FIGURE 1** Soils samples collected from experimental field and their relative GPS coordinates.

**SUPPLEMENTAL TABLE 1** Chemical analysis of fertilizers composition: (**HP1**) Hydrolysate of proteins 1; (**HP2**) Hydrolysate of proteins; (**L**) Leather; (**R**) Rhizovit; (**S**) Synthesis.

|  | **HP1** | **HP2** | **L** | **R** | **S** | |
| --- | --- | --- | --- | --- | --- | --- |
|  | **Saprid forte 9%, ILSA SPA** | **Protifert 8.3, SICIT 2000 SPA** | **Leather meal, ILSA SPA** | **Rhizovit N20, TIMAC AGRO** | **Urea^a^** | **Ammonium nitrate^b^** |
| **Organic nitrogen %** | **9.0** | **8.3-9** | **12.5** | **--** | **--** | **--** |
| **Total nitrogen. %** | **9.0** | **9** | **12.5** | **20.0** | **46.0** | **32.00** |
| **Ammonia nitrogen. %** | **--** | **0.7** | **--** | **12.0** | **--** | **16.00** |
| **Nitric nitrogen %** | **--** | **--** | **--** | **--** | **--** | **16.00** |
| **Ureic nitrogen %** | **--** | **--** | **--** | **8.0** | **46.0** | **--** |
| **Organic carbon %** | **24.7** | **26** | **41.0** | **--** | **--** | **--** |
| **pH of 10% solution in water** | **5.3** | **6-7** | **4.5** | **--** | **--** | **--** |
| **Ashes %** | **4.4** | **7.7** | **--** | **--** | **--** | **--** |
| **Total amminoacids %** | **57.0** | **52.0** | **--** | **--** | **--** | **--** |
| **Calcium %** | **--** | **0.2** | **--** | **--** | **--** | **--** |
| **Chloride %** | **--** | **3.3** | **--** | **--** | **--** | **--** |
| **Sulfate%** | **--** | **1.0** | **--** | **--** | **--** | **--** |
| **Sodium %** | **--** | **2.2** | **--** | **--** | **--** | **--** |
| **Salinity dS/m** | **0.60** | **--** | **--** | **--** | **--** | **--** |
| **Sulfuric anhydride H_2_O % solution** | **--** | **--** | **--** | **30.0** | **--** | **--** |
| **Total Iron %** | **--** | **--** | **--** | **0.2** | **--** | **--** |

*^a,b^fertilizers used together.*

**SUPPLEMENTAL TABLE 2** Physical, chemical and microbiological properties of soil samples collected from experimental field, as reported in Supplemental Figure 1. Further details were reported in Materials and methods.

|  |  | ***Soil sampling*** | | | | | | | |
| --- | --- | --- | --- | --- | --- | --- | --- | --- | --- |
| **Parameters** | **Measure Unit** | **camp.1** | **camp.2** | **camp.3** | **camp.4** | **camp.5** | **camp.6** | **camp.7** | **camp.8** |
| **Sand** | % | 45 | 36 | 45 | 46 | 46 | 49 | 50 | 49 |
| **Silt** | % | 22 | 26 | 22 | 21 | 22 | 20 | 19 | 20 |
| **Clay** | % | 33 | 38 | 33 | 33 | 33 | 31 | 31 | 31 |
| **Soil texture** |  | Clay loam | Clay loam | Clay loam | Sandy clay loam | Sandy clay loam | Sandy clay loam | Sandy clay loam | Sandy clay loam |
| **pH** |  | 7 | 7 | 7.1 | 7 | 7 | 7.2 | 7 | 7 |
| **Soil EC^a^** | mS | 0.148 | 0.146 | 0.144 | 0.199 | 0.138 | 0.125 | 0.093 | 0.106 |
| **Total limestone** |  | *traces* | *traces* | *traces* | *traces* | *traces* | *traces* | *traces* | *traces* |
| **SOM^b^** | % | 1.97 | 2.03 | 1.76 | 1.45 | 1.27 | 1.25 | 1.45 | 1.63 |
| **Total nitrogen** | % | 0.111 | 0.113 | 0.100 | 0.092 | 0.082 | 0.081 | 0.092 | 0.100 |
| **Phosphorus (available)** | mg/kg | 24 | 21 | 21 | 25 | 26 | 19 | 19 | 19 |
| **Iron (available)** | mg/kg | 18.6 | 21.6 | 21.2 | 21.6 | 22.2 | 19.6 | 20.4 | 19.8 |
| **Manganese (available)** | mg/kg | 12 | 13.4 | 11.4 | 11.8 | 13.2 | 10.6 | 11.4 | 11.8 |
| **Copper (available)** | mg/kg | 3 | 3 | 3 | 3.2 | 2.8 | 3 | 2.8 | 2.6 |
| **Zinc (available)** | mg/kg | 2.2 | 2 | 2 | 2.2 | 2.2 | 2.8 | 2.4 | 1.9 |
| **Boron (soluble)** | mg/kg | 1.12 | 1.36 | 1.06 | 1.02 | 0.92 | 0.90 | 1.18 | 0.90 |
| **Calcium (exchangeable)** | mg/kg | 3200 | 3700 | 3500 | 3100 | 2950 | 2950 | 3100 | 2900 |
| **Magnesium (exchangeable)** | mg/kg | 232 | 276 | 188 | 196 | 194 | 188 | 176 | 208 |
| **Potassium (exchangeable)** | mg/kg | 759 | 778 | 656 | 626 | 657 | 641 | 558 | 645 |
| **Sodium (exchangeable)** | mg/kg | 62 | 64 | 54 | 60 | 41 | 53 | 76 | 101 |
| ***C.E.C analysis*** |  |  |  |  |  |  |  |  |  |
| **C.E.C^c^** | mEq | 20.14 | 23.07 | 20.98 | 18.99 | 18.23 | 18.19 | 18.73 | 18.32 |
| **Calcium** | mEq | 16 | 18.50 | 17.50 | 15.50 | 14.75 | 14.75 | 15.50 | 14.50 |
| **Magnesium** | mEq | 1.93 | 2.30 | 1.57 | 1.63 | 1.62 | 1.57 | 1.47 | 1.73 |
| **Potassium** | mEq | 1.94 | 1.99 | 1.68 | 1.60 | 1.68 | 1.64 | 1.43 | 1.65 |
| **Sodium** | mEq | 0.27 | 0.28 | 0.23 | 0.26 | 0.18 | 0.23 | 0.33 | 0.44 |
| **Basic saturation** | mEq | high | high | high | high | high | high | high | high |
| **Mg/K ratio** | mEq/mEq | 1.0 | 1.2 | 0.9 | 1.0 | 1.0 | 1.0 | 1.0 | 1.0 |
| **Microbiological results: Biolog Ecoplate** | Average Well Color Development (AWCD)^d^ | 1.58 | 1.59 | 1.43 | 1.34 | 1.39 | 1.37 | 1.19 | 1.21 |

*^a^Soil electrical conductivity.*

*^b^Soil organic matter.*

*^c^Cation-exchange capacity of soil.*

*^d^Optical density measured at 590 nm.*

**SUPPLEMENTAL TABLE 3** Climatic data of the sampling area for the three-year period 2010-2012. Data were downloaded from climate station (Roma Flaminio) close to sampling site. Download site: http://www.idrografico.roma.it/annali/

|  |  | **2010** | **2011** | **2012** |
| --- | --- | --- | --- | --- |
| ***Temperature (°C)*** | ***Jan*** | *7,9* | *8,7* | *8* |
|  | ***Feb*** | *9,3* | *9,2* | *5,9* |
|  | ***Mar*** | *11,2* | *11,3* | *13,7* |
|  | ***Apr*** | *14,8* | *16* | *14,6* |
|  | ***May*** | *17,8* | *19,4* | *17,9* |
|  | ***Jun*** | *22,3* | *23,4* | *23,9* |
|  | ***Jul*** | *26,9* | *24,2* | *26,4* |
|  | ***Aug*** | *25,2* | *26,1* | *27,3* |
|  | ***Sep*** | *21,2* | *23,8* | *21,9* |
|  | ***Oct*** | *16,9* | *17,4* | *18,6* |
|  | ***Nov*** | *13,1* | *12,8* | *14,4* |
|  | ***Dec*** | *8,5* | *10,1* | *7,8* |
| ***Rainfall (mm)*** | ***Jan*** | *107,8* | *50,6* | *29,6* |
|  | ***Feb*** | *126,4* | *42,8* | *127,2* |
|  | ***Mar*** | *65,6* | *120* | *4* |
|  | ***Apr*** | *74,4* | *28,8* | *60,8* |
|  | ***May*** | *75,8* | *35,2* | *70,2* |
|  | ***Jun*** | *37* | *8,6* | *0* |
|  | ***Jul*** | *33,4* | *59,6* | *5,8* |
|  | ***Aug*** | *4,4* | *0* | *32,4* |
|  | ***Sep*** | *14,4* | *16,8* | *86,4* |
|  | ***Oct*** | *29,4* | *86,2* | *103,2* |
|  | ***Nov*** | *284* | *41* | *85* |
|  | ***Dec*** | *122,2* | *45,4* | *90,4* |

**SUPPLEMENTAL TABLE 4** List of primer pairs used for qPCR analysis of gene expression.

| **Protein name** | **Gene name** | **AGI code^b^** | **Forward primer** | **Reverse Primer** |
| --- | --- | --- | --- | --- |
| ***Primers designed from RNA-SEQ data^a^*** |  |  |  |  |
| Polyubiquitin 10 (housekeeping gene) | *UBQ10* | AT4G05320.2 | 5’-tggcatgcagatcttcgtca-3’ | 5’-ctggatcttcgccttgacgt-3’ |
| NADH-dependent glutamate synthase 1 | *GLT1* | AT5G53460.1 | 5’-agaggtgacaaaggatgccg-3’ | 5’-cttctcatcggttggcagga-3’ |
| Glutamate dehydrogenase 1 | *GDH1* | AT5G18170.1 | 5’-tgggctgctcaactgatcact-3’ | 5’-ttggagttcttgacagcccc-3’ |
| Glutamate dehydrogenase 2 | *GDH2* | AT5G07440.1 | 5’-cagcttctggatcggttgga-3’ | 5’-gcggggttcttaacagggaa-3’ |
| Citrate synthase 2 | *CSY2* | AT3G58750.1 | 5’-catccccaccctaccaacac-3’ | 5’-tggagcccttggttgttgtt-3’ |
| Citrate synthase 3 | *CSY3* | AT2G42790.1 | 5’-gatgcgatttatgtgcgtgg-3’ | 5’-ggaacatcaccgaaagagcag-3’ |
| Citrate synthase 4 | *CSY4* | AT2G44350.2 | 5’-ccccatgagactccaggttg-3’ | 5’-cccaactgctccgatgtagg-3’ |
| NAD-dependent malate dehydrogenase 2 | *C-NAD-MDH2* | AT5G43330.1 | 5’-caggcaaaaggggagctaca-3’ | 5’-tctctacccagaacagccgc-3’ |
| Nitrate transporter 1.2 | *NRT1.2* | AT1G69850.1 | 5’-cgtgcgtgtgtgtgtgaactc-3’ | 5’ ctgagagccttcctgaacgg-3’ |
| Nitrate reductase 1 | *NR1* | AT1G77760.1 | 5’-ggatcacgcccatgtaccag-3’ | 5’-tgcatctccgtctcgtcctc-3’ |
| Nitrate reductase 2 | *NR2* | AT1G37130.1 | 5’-gcgttgatggggaggatctc-3’ | 5’-cgccccgagtacatcatcaa-3’ |
| Ferredoxin nitrite reductase | *NIR1* | AT2G15620.1 | 5’-cgctcccacttcttgtcgat-3’ | 5’-tcgagaagaggatgccgaac-3’ |
| Glutamine-dependentasparagine synthase 1 | *ASN1* | AT3G47340.1 | 5’-atcacgtgcagcaatgaagc-3’ | 5’-tacgaggagcatggggagaa-3’ |
| Aspartate aminotransferase | *AAT* | AT2G22250.2 | 5’-caagccctgccttctgtgat-3’ | 5’-gactggttggcgacttggat-3’ |
| Glutamate synthase 1 | *GLU1* | AT5G04140 | 5’-caaacaggcaaactgatgggt-3’ | 5’-gccatccaccagcatttga-3’ |
| Glutamate synthase 2 | *GLU2* | AT2G41220 | 5’-taacgtcaccaaaggctgca-3’ | 5’-gacggtatctgcaagctcgtt-3’ |
| ***Primers designed from multialignment*** |  |  |  |  |
| Nitrate transporter 2.3 | *NRT2.3* | AT5G60780.1 | 5’-tggactcggagcacaaggc-3’ | 5’-gagacgaagcaggtgaagaagg-3’ |
| Nitrate transporter 2.6 | *NRT2.6* | AT3G45060.1 | 5’-acatgcgcaccttccacc-3’ | 5’-gaggttgtcgcggatgatg-3’ |
| Citrate synthase 1 | *CSY1* | AT3G58740.1 | 5’-gcaccttgcttcaagtggtgt-3’ | 5’-agtactgcctcatttgcaccac-3’ |
| Isocitrate dehydrogenase [NAD] subunit 2 | *IDH2* | AT2G17130.1 | 5’-gcgccggtctacttcgagac-3’ | 5’-ccttgaggcagaccttgttgc-3’ |
| ***Primers designed from Triticum* sequence** |  |  |  |  |
| GS2 plastid glutamine synthetase isoform | *GS2a* | DQ124212.1* | 5’-agtacactgagcatgcgcgaag-3’ | 5’- tgtctcgtgtagccctgtcaac-3’ |

*^a^As reported in Supplemental Note 1.*

*^b^Identifiers correspond to the AGI code of the closest Arabidopsis thaliana homologous gene identified by blast search, except for GS2a, for which the GenBank accession number is provided.*

| **Spot No. (a)** | **Relationship list (b)** | **Acc. No. (c)** | **Protein Name** | **Organism** | **Protein Length (d)** | **E-Value (e)** |
| --- | --- | --- | --- | --- | --- | --- |
| **4** | **8** | **A0A1C7D3S9** | **Ribulose bisphosphate carboxylase large chain** | *Robinia pseudoacacia* | 482 | 9.1e^-128^ |
| **4** | **10** | **A0A1C6ZYA3** | **Cytosolic small heat shock protein 17.5** | Elymus sibiricus | 158 | 3.4e^-102^ |
| **5** | **11** | **M8AVR4** | **20 kDa chaperonin, chloroplastic** | *Aegilops tauschii* | 247 | 1.4e^-149^ |
| **6** | **15** | **Q93Y73** | **Aspartate-semialdehyde dehydrogenase family protein, expressed** | *Oryza sativa subsp. japonica* | 375 | 0.0 |
| **6** | **16** | **A0A1E5UJ66** | **3-hydroxyisobutyryl-CoA hydrolase-like protein 3, mitochondrial** | *Dichanthelium oligosanthes* | 410 | 0.0 |
| **8** | **19** | **M8AW52** | **Quinone oxidoreductase-like protein** | *Aegilops tauschii* | 309 | 0.0 |
| **9** | **23** | **A0A1E5UYJ2** | **Putative aldo-keto reductase 2** | *Dichanthelium oligosanthes* | 346 | 0.0 |
| **9** | **24** | **A0A1E5V2P1** | **Chloroplast stem-loop binding protein of 41 kDa a, chloroplastic** | *Dichanthelium oligosanthes* | 437 | 1.7e^-120^ |
| **10** | **28** | **M8AW52** | **Quinone oxidoreductase-like protein** | *Aegilops tauschii* | 309 | 0.0 |
| **10** | **30** | **M7ZG23** | **Cell division cycle protein 48-like protein** | *Triticum urartu* | 818 | 0.0 |
| **12** | **35** | **A0A1E5VC92** | **50S ribosomal protein L1, chloroplastic** | *Dichanthelium oligosanthes* | 348 | 0.0 |
| **12** | **37** | **A0A0U2KWW3** | **Malate dehydrogenase** | *Hordeum vulgare* | 395 | 0.0 |
| **12** | **40** | **Q69LA6** | **Probable pyridoxal 5'-phosphate synthase subunit PDX1.1** | *Oryza sativa subsp. japonica* | 318 | 8.8e^-132^ |
| **13** | **42** | **D7MSY9** | **Thylakoid lumenal 17.4 kDa protein, chloroplast** | *Arabidopsis lyrata subsp. lyrata* | 236 | 1.5e^-80^ |
| **18** | **51** | **Q10G56** | **Ornithine aminotransferase, mitochondrial** | *Oryza sativa subsp. japonica* | 473 | 0.0 |
| **18** | **52** | **A0A1C7D3S9** | **Ribulose bisphosphate carboxylase large chain** | *Robinia pseudoacacia* | 482 | 9.1e^-128^ |
| **20** | **56** | **Q6IY71** | **Mitochondrial ATP synthase** | *Triticum aestivum* | 238 | 2.8e^-143^ |

**SUPPLEMENTAL TABLE 5** Additional information by blast analysis related to the uncharacterized and predicted proteins reported in Table 3.

*^a^Spot numbers correspond to those reported in Figure 3.*

*^b^numbers correspond to those reported in Table 3.*

*^c^UniProtKB, accession number.*

*^d^protein size (aa) as reported in the blast output.*

*^e^expected value, from blast analysis.*

**SUPPLEMENTAL TABLE 6** 2^-ΔΔCt^ values of the genes analyzed *via* qPCR. Mean values ± standard deviation (n=3) were represented for each gene separately, through a colorimetric scale: minimum (**red**, low relative expression) and maximum (**green**, high relative expression) value through black (midpoint). Sample names and gene identities are specified in Figure 1B and Supplemental Table 4, respectively. Control samples are marked in red.

|  | **CC** | **CL** | **CR** | **CS** | **CHP1** | **CHP2** | **DC** | **DL** | **DR** | **DS** | **DHP1** | **DHP2** |
| --- | --- | --- | --- | --- | --- | --- | --- | --- | --- | --- | --- | --- |
| ***GLT1*** | 1 ± 0,07 | 19,58 ± 3,71 | 8,73 ± 0,75 | 8,51 ± 1,24 | 9,16 ± 0,24 | 3,75 ± 0,17 | 7,49 ± 0,26 | 21,57 ± 5,18 | 5,94 ± 0,23 | 7,65 ± 2,34 | 81,17 ± 4,10 | 6,55 ± 1,05 |
| ***GDH1*** | 1 ± 0,12 | 2,39 ± 0,43 | 1,42 ± 0,16 | 1,96 ± 0,56 | 0,99 ± 0,07 | 2,01 ± 0,14 | 1,6 ± 0,1 | 1,05 ± 0,16 | 1,14 ± 0,06 | 2,03 ± 0,61 | 5,22 ± 0,24 | 3,14 ± 0,5 |
| ***GDH2*** | 1 ± 0,04 | 5,3 ± 1,12 | 2,78 ± 0,13 | 30,18 ± 4,87 | 0,92 ± 0,03 | 1,5 ± 0,15 | 2,38 ± 0,49 | 26,34 ± 2,19 | 1,58 ± 0,13 | 3,24 ± 0,98 | 2,76 ± 0,05 | 3,24 ± 0,53 |
| ***NR1*** | 1 ± 0,18 | 0,17 ± 0,23 | 0,26 ± 0,04 | 0,35 ± 1,91 | 0,14 ± 0,48 | 0,14 ± 0,08 | 0,13 ± 0,03 | 0,04 ± 0,01 | 0,16 ± 0,33 | 0,61 ± 1,41 | 0,26 ± 0,49 | 0,06 ± 0,11 |
| ***NR2*** | 1 ± 0,04 | 0,46 ± 0,08 | 0,12 ± 0,02 | 0,12 ± 0,02 | 0,31 ± 0,04 | 0,16 ± 0,01 | 0,54 ± 0,03 | 0,18 ± 0,03 | 0,29 ± 0,13 | 0,38 ± 0,14 | 0,44 ± 0,01 | 0,09 ± 0,01 |
| ***CSY1*** | 1 ± 0,5 | 6,21 ± 1,52 | 0,8 ± 0,235 | 9,28 ± 1,46 | 1,19 ± 0,14 | 0,69 ± 0,21 | 4,38 ± 0,79 | 3,4 ± 0,27 | 2,28 ± 0,47 | 2,6 ± 0,89 | 4,3 ± 1,00 | 0,82 ± 0,13 |
| ***CSY2*** | 1 ± 0,27 | 4,07 ± 1,49 | 0,52 ± 0,15 | 5,45 ± 0,71 | 1,09 ± 0,03 | 0,33 ± 0,15 | 3,87 ± 0,75 | 4,53 ± 0,1 | 1,61 ± 0,21 | 5,8 ± 1,72 | 10,99 ± 0,88 | 3,15 ± 0,80 |
| ***CSY3*** | 1 ± 0,63 | 3,95 ± 1,22 | 2,5 ± 0,13 | 1,29 ± 1,13 | 1,06 ± 0,09 | 1,86 ± 0,17 | 2,03 ± 0,05 | 1,81 ± 0,99 | 1,05 ± 0,02 | 2,78 ± 0,83 | 3,55 ± 0,45 | 5,2 ± 0,87 |
| ***CSY4*** | 1 ± 0,34 | 1,76 ± 1,24 | 1,74 ± 0,09 | 1,28 ± 0,25 | 1,12 ± 0,29 | 2,31 ± 0,36 | 2,97 ± 0,70 | 1,54 ± 0,32 | 0,69 ± 0,11 | 4,62 ± 1,45 | 10,26 ± 1,85 | 8,77 ± 1,71 |
| ***MDH2**** | 1 ± 0,08 | 0,45 ± 0,08 | 0,28 ± 0,02 | 0,78 ± 0,10 | 0,56 ± 0,04 | 0,56 ± 0,03 | 0,95 ± 0,01 | 0,54 ± 0,02 | 0,33 ± 0,01 | 1,84 ± 0,55 | 4,46 ± 0,05 | 5,68 ± 1,08 |
| ***NRT1.2*** | 1 ± 0,06 | 2,28 ± 0,65 | 1,03 ± 0,05 | 5,41 ± 0,705 | 1,06 ± 0,15 | 0,32 ± 0,02 | 1,66 ± 0,22 | 1,9 ± 0,33 | 1,18 ± 0,12 | 5,31 ± 1,57 | 4,39 ± 0,04 | 1,94 ± 0,35 |
| ***NRT2.3*** | 1 ± 0,10 | 9,99 ± 1,83 | 1,68 ± 0,22 | 7,84 ± 1,4 | 0,91 ± 0,07 | 0,32 ± 0,01 | 1,88 ± 0,55 | 2,03 ± 0,77 | 1,85 ± 0,65 | 2,1 ± 0,84 | 2,05 ± 0,51 | 0,04 ± 0,01 |
| ***NRT2.6*** | 1 ± 0,04 | 6,83 ± 1,62 | 5,11 ± 1,19 | 7 ± 1,465 | 0,86 ± 0,05 | 0,26 ± 0,06 | 1,98 ± 0,31 | 2,08 ± 0,13 | 1,45 ± 0,06 | 1,78 ± 0,77 | 1,6 ± 0,08 | 0,05 ± 0,03 |
| ***IDH2*** | 1 ± 0,03 | 1,44 ± 0,27 | 0,63 ± 0,03 | 1,96 ± 1,05 | 0,76 ± 0,06 | 0,44 ± 0,06 | 0,99 ± 0,16 | 1,48 ± 0,05 | 0,62 ± 0,2 | 1,34 ± 0,41 | 1,27 ± 0,22 | 0,34 ± 0,41 |
| ***ASN1*** | 1 ± 0,49 | 4,79 ± 0,37 | 3,6 ± 0,57 | 46,37 ± 11,65 | 1,03 ± 0,13 | 1,43 ± 0,34 | 2,54 ± 0,74 | 50,76 ± 1,1 | 0,76 ± 0,21 | 7,05 ± 1,79 | 2,72 ± 1,25 | 15,29 ± 5,79 |
| ***AAT*** | 1 ± 0,53 | 1,89 ± 0,69 | 0,53 ± 0,21 | 1,88 ± 0,51 | 1 ± 0,28 | 0,43 ± 0,16 | 1,79 ± 0,49 | 2,16 ± 0,24 | 0,65 ± 0,16 | 1,92 ± 1,28 | 8,19 ± 2,14 | 2,68 ± 1,37 |
| ***NIR1*** | 1 ± 0,55 | 1,06 ± 0,45 | 0,11 ± 0,02 | 0,85 ± 0,11 | 0,26 ± 0,03 | 0,09 ± 0,05 | 0,53 ± 0,14 | 0,41 ± 0,01 | 0,57 ± 0,15 | 0,65 ± 0,17 | 0,4 ± 0,11 | 0,06 ± 0,03 |
| ***GLU1*** | 1 ± 0,09 | 0,23 ± 0,03 | 0,22 ± 0,02 | 0,03 ± 0,01 | 0,16 ± 0,02 | 0,35 ± 0,07 | 0,64 ± 0,13 | 0,02 ± 0,01 | 0,17 ± 0,03 | 0,05 ± 0,01 | 0,45 ± 0,18 | 0,38 ± 0,03 |
| ***GLU2*** | 1 ± 0,07 | 0,16 ± 0,02 | 0,12 ± 0,01 | 0,08 ± 0,01 | 0,16 ± 0,02 | 0,32 ± 0,03 | 0,77 ± 0,09 | 0,07 ± 0,01 | 0,22 ± 0,04 | 0,07 ± 0,01 | 0,44 ± 0,05 | 0,33 ± 0,01 |
| ***GS2A*** | 1 ± 0,16 | 1,3 ± 0,37 | 1,25 ± 0,33 | 0,34 ± 0,09 | 1,8 ± 0,7 | 0,45 ± 0,18 | 1,79 ± 0,47 | 1,8 ± 0,16 | 0,34 ± 0,06 | 1,11 ± 0,46 | 1,04 ± 0,37 | 1,31 ± 0,17 |

**SUPPLEMENTAL NOTE 1 Sequences from** [**Krasileva et al. (2013)**](#_ENREF_1) **used for primer design**

**>polyubiquitin_10**

CAAGGAGGGCATCCCACCGGACCAGCAGCGCCTCATCTTTGCTGGTAAGCAGCTTGAGGACGGCCGCACCCTCGCTGACTACAACATCCAGAAGGAGTCCACCCTCCACCTGGTGCTCCGTCTCAGGNNNNNNNNNNNNNNNNNNNNNNNNNNNNNNNNNNNNNNNNNNNNNNNNNGTGCTCCGCCTCAGGGGTGGCATGCAGATCTTCGTCAAGACCCTCACCGGCAAGACCATCACCCTTGAGGTCGAGTCCTCTGACACGATCGACAACGTCAAGGCGAAGATCCAGGACAAGGAGGGCATCCCTCCGGACCAGCAGCGCCTCATCTTCGCTGGCAAGCAGCTTGAGGACGGTCGCACCCTCGCTGACTACAACATCCAGAAGGAGTCCACC

**>glutamate_synthase_1_[NADH]**

GAAAAACACTGGTGATGGTGCCGGCATTCTCGTTGCTCTACCACACACCTTCTTCCGAGAGGTGACAAAGGATGCCGGTTTCGAGTTACCGCCACCAGGTGAGTATGCTGTTGGAATGGTCTTCCTGCCAACCGATGAGAAGCGTCGCGAGAGGAGCAAAACTGAGTTTACAAAGGTCGCGGAGTCGCTAGGACATTCGATACTTGGGTGGCGCCAGGTTCCCACTGACAATTCAGACTTGGGCCAAGCTGCTCTCGACACTGAACCAGCGATTGAACAGGTTTTCCTCACCAAGAGTCCAAACTCGAAGGCCGACTTCGAACAGCAGTTGTTTATCCTGAGGAGGCTTTCAATTGTATCTATCCGGGCCGCGCTGAATCTCAAGCGTGGAGGAGAGAGAGATTTCTACATGTGCTCTTTATCTTCAAGGACCATTGTCTACAAGGGCCAGCTTATGCCGTCTCAGCTTCAGGGGTACTACTATGCGGACATAGGTTCACTCAAGGTTCTCCACCAACACCT

**>glutamate_dehydrogenase_1**

CAGCATGACAATGCCAGGGGGATAGTCTTTGTGCCGCTGCTCGCCACTAACACTTCGCGGCTACTCCACTCGCCGGCGCGGCGGCTCCGGAGGGGGGATTTCGTCGGGCAGGATGAACGCATTGGCGGCCACCAGCCGGAACTTCAAGCAGGCGGCCAAGCTGCTGGGCCTCGACTCCAAGCTCGAGAAGAGCCTGCTCATCCCCTTCAGGGAGATCAAGGTTGAGTGCACAATCCCGAAAGATGATGGGACATTAGCATCCTATGTTGGGTTTAGGGTGCAGCATGACAATGCCAGGGGCCCTATGAAGGGTGGAATCAGATACCACCATGAGGTTGATCCTGACGAGGTCAATGCCTTGGCGCAACTGATGACATGGAAAACAGCCGTGGCCAATATTCCGTATGGAGGCGCTAAAGGTGGCATTGGGTGCAGTCCCGGAGACCTGAGCATATCGGAGCTCGAACGGCTTACCCGAGTTTTCACCCAGAAAATTCATGACTTAATTGGCATCCACACCGATGTTCCAGCTCCAGATATGGGCACCAACGCACAGACAATGGCATGGATACTGGATGAGTACTCAAAGTTCCATGGCTACTCACCTGCTGTGGTGACAGGAAAGCCTGTGGACCTTGGAGGATCACTGGGAAGAGATGCAGCTACTGGAAGGGGAGTTCTGTTTGCCACTGAAGCCCTACTTGCAGAGCATGGCAAAGGCATTGCAGGCCAGCGTTTTGTAATCCAGGGATTCGGTAATGTTGGCTCCTGGGCTGCTCAACTGATCACTGAAGCTGGCGGCAAGGTGATCGCCATCAGCGATGTCACAGGGGCTGTCAAGAACTCCAATGGCATTGACATAGCCAAGCTGATGAAGCACTCGGCGGAGAACCGTGGGATCAAGGGCTTTGACGGAGGAGACGCCGTCGACCCGGCCTCGCTGCTCACCGAAGAGTGCGATGTGCTCATCCCGGCAGCGCTGGGAGGAGTCATAAACAAGGACAATGCTGATGCCATCAAAGCAAAATACATCATCGAGGCTGCGAACCACCCGACAGACCCCGAGGCCGACGAGATTCTGGCGAAGAAGGGGGTGCTGATCCTGCCGGACATCCTGGCCAACTCGGGTGGCGTGACGGTGAGCTACTTCGAGTGGGTGCAGAACATCCAGGGGTTCATGTGGGACGAGGAGAAGGTGAACCGGGAGCTCAAGACGTACATGACCCGCGCCTTCCGGGACACCAAGGAGATGTGCCGCTCCCACCACTGCGACCTCCGCATGGGCGCCTTCACCCTCGGCGTCAACCGCGTCGCCCGCGCCACCGTCCTCCGCGGCTGGGAGGCCTGATCGACCGCCGGCCGGCATCCTGCAATGCCCGTGCTCTCTCGCCGTCTCCATGGGGATCCCGTCCAAGTCTCAACTCAACAAAAAGTTCAATAATCGAGCCCGTGTGTTCTCTCATACATAAGCTCCGATCTCTTGGAGAGACCGGCAGAACAGCAGCAGCAGCACCTTCTTCTTGTAATCCGCGGCTGAGAGTGAAACACAGCAATGTAATTCGGTTTCTTTTTCTTGGTAACATTTGATCGTGTTGATGATGCGTGAGTCGATTTTATTAGCATTTCG

**>glutamate_dehydrogenase_2**

CCCTGCCATAAGTTGCTGAAACTCTCAATTGCCTGAATCTTGCAAAAGTAATGTATTACATTTGGGGCCATCAAATCTCAGACCAGAAATGTTGAAACCAATGAGGCCAAAATAACCTTTTTGATATTAAAATCCAGAAACTTTTGGTAAACATTCAACAAATCTGAAGGTTTACAATCACACAGAACTAGATCACATAGTTGGCAACCCAGGGCAGATAATTAGCTAACAGAAAGAACAAACCCATCGTCTCTGTCATGTACACCGAAAATTATTTCCCATGCCGGCAACTTATGATGAATTTACCAAATCAATGATGGTGGATATAAGAACTTTCATCCACCACGCTGCCGGGCATGGATTATTCAATAAGGAATCTGGAATTCCTCATGCCTCCCAACCCCTCAAGATGGTGGCGCGAGCAACCCGGTTCACTCCCAAGGTGAATGCTCCCATCCTAAGGNNTTCTTCTTCCCACATGAATCCTTGAATGTTCTGAACCCACTCAAAATAGCTAACGATCACACCCCCAGCATTAGCATAGATATCAGGTAACACGACCACTCCCTTCTTGGTAAGAATCTCATCAGCTTCTGGATCGGTTGGATGATTAGCAGCTTCGATTATAAATTTGGCCTTAACATCAGGGGCATTTTCCCTGTTAAGAACCCCGCCTAAGGCACATGGGAGGAGGACATCGCATTCATGCACTAGCAACTCTGAGGCATCCATG

**>citrate_synthase_2**

CGACGAGTAAATCGGAACGGAGGGAGTACAATGCATCCATCCCCACCCTACCAACACAGCACATCAATTGTCAATACAAGTAACTAATATCGACAAACAACAACCAAGGGCTCCATCCATCATCATGCCCTCGGTCTTCTTCGGTCACACCATAAGCAAACAAGAGCCCATTCCTGAATACTACAAACATCAGCTCACAGACTAAAACGGTTCTCCTCCATTGCTAAAACAACCAATCGAAACACTCTGGAGTCCCAGGCTTCTTCCTTCAACTCACCAACGAAAATCTTTCTCCTACAGCGACGCCAAAAACCTTCCTCCACTTACATAGCACCTTCTGTGGGTAGTCATGGTGGACGGCCTCATAGTGGAGGTTGAGGTCGGAGTCGACGATGTCATCGTCAGCGTGGGTGCATGTCGACACCATCAATGTCATCCATGGAACTGGAGGGCGCCTGTTAAAGCAGAACGGATGTAGAGTGAGGGCCACTGAAAAGGTTGATGCATGCTGAACAGGAAGGAGCAGATCCAGCACTCGCTGTCGAGGCCTCATAATTTAATTGTCAGGGTCATCAAGTGACGCCTTCCAATGTGCTAACCAACCGGCCATGCGAGGAATTGCAAATAGCACAGGGAAATTTCTGTAGGGAACCCCATTGCTCTATATATCAGGCCAGAGTAAAAATCCACATCTGGATACAGCTTCCTCTTGATAAAATACTCGTCTGACAGTGCTGCCTTCTCCAAAACAACATCAACCTCATTTGCGCCACCATGAAGTGGACCATATAGAGCTCCAACAACACCAGAAAGAGCAGTTTAGACATCGACACCACCATATAGATATCATAGCAAACCATGATATCCAGTTGTTAAACAGACCCTAACCATACACATGCAACCCCTAGATGAGAAGAGCCTGACACGTTACTCTGAACCTGAAATCAACAGGCACGAGTTAGTTGTGCTCTAGACCAGAAAAGGCATAAAGAATTCATGAGTATCAGAATGTTGAATAGTAAATACTTGGGTATCAGTTCGTCAACGACATCAGTTGGACTAATATGCAGAAATTGTAGATGAATGGCATTGACTAGCATATTAAATGGATGACACTAGGGTCATAGAAAGAGTGCCAGGTCATAAGGAAATAACACAGGGAACAGGAAAAGGAAAATCAACAAAA

**>citrate_synthase_3**

GCCGCGCTGGGGAGGTCGCCGGCGTCGGCCGCGGCCCCGGGGGCCCGCGCCGGCGTGCTCGCCGTGGTGGACTCGAGGACCGGGAAGCGGTACGAGGTCAAGGTTTCGGAGGACGGCACCGTCCGCGCCACAGACTTCAAGAAGATTACCACTGGGAAAGATGACAAGGGTCTTAAGACTTACGATCCTGGTTATCTCAACACTGCCCCTGTGCGTTCTTCCATCTGCTACATTGATGGGGATGAGGGAATTCTTCGCTACAGGGGTTATCCAATTGAGGAGGTGGCCGAAAGCAGTTCGTTTGTTGAGGTCGCCTACCTCTTAATGTACGGGAACTTGCCCACTCAGAGTCAACTGGCAGGCTGGGAATTTGCAATTTCACAGCACTCTGCTGTTCCTCAAGGACTCTTGGATATCATACAATCAATGCCTCACGATGCCCACCCCATGGGTGTCCTTGCCAGTGCAATGAGCACACTTTCTGTCTTCCACCCAGATGCAAACCCTGCCCTTAGAGGGCAAGATCTGTACAACTCAAAGCAGGTTAGGGATAAGCAAATTGTGCGAGTTCTTGGGAAGGCACCAGCAATAGCAGCTGCAGCCTACCTGAGACTAGCAGGAAGGCCTGCTGTCCTTCCTTCAAATAATCTTTCTTATTCAGAGAATTTCTTGTATATGCTAGACTCTTTGGGTAACAAAGAATATAAGCCAAATCCCCGACTTGCACGGGTTCTAGATATCCTTTTTATTCTCCATGCTGAACACGAAATGAACTGCTCAACAGCTGCTGTTAGGCACCTTGCTTCAAGTGGTGTCGATGTCTTCACTGCTCTTTCTGGCGCTGTTGGAGCTCTATATGGTCCACTGCATGGTGGCGCAAATGAGGCGGTACTTAAAATGTTGAATGAGATTGGAGCTGTGGAGAATATTCCAGATTTCATTGAGGGAGTGAAGAACAGGAAACGGAAAATGTCAGGTTTTGGGCACCGTGTGTATAAGAATTATGATCCTCGTGCTAAAGTCATCCGAAAGTTAGCAGAGGAGGTTTTCTCTATTGTTGGACGGGATCCTCTTATCGAGGTTGCTGTTGCTTTGGAGAAGGCAGCACTGTCAGACGATTATTTTATCAAGAGGAAGCTGTATCCAAATGTGGATTTTTACTCAGGCCTGATTTACAGAGCGATGGGATTCCCTACAGAATTTTTCCCTGTGTTATTTGCAATTCCTCGCATGGCTGGTTGGCTAGCACATTGGAAGGAGTCACTTGATGACCCTGACAATAAAATTATGAGGCCTCAACAGGTATACACGGGCGTTTGGCTGAGGCACTACACCCCTGTGAGGGAACGAGTGGCATCTAACCAGGGCGAGGAGCTCGGTCAGATCGCTGCATCAAACGCAACGAGGCGTCGCCGTGCTGGTTCTTCCCTGTAGAACAGCAGCAGGCACGGATGCATGGTGCAGCATCCAGCCCACGCAATAAACCAGGCTGCTGTCGTTGCCCCTCACAACGCCGCAGCGGCTCGAGTCCGGGAGCTGTTATCCTTCTGGTGTCACCAAGACACTTAGCAGTTGCCATGCAATAAACTTCTAGTAAAAGGCACGCTGTGGACACTATCTATGCACACACTTCCAGTTTCCAGCTTCATCTCTGCATGCATGCAATGTGAGCTCATGATTGGAAAAAATAATCATACTCCCTCCGTCCCAAAATAAGTCTTAACTTTAGAGGGAGTAGTTGATGGCATGTCGGTCTTCGTAAGAGTGTGCATATTAGTGCGGTGCGTAGGATTCGATCCCTGGCCAGCAATGAAAAATCATGCCATTTGACCGTTAGTTAACGCGCTATTTAGTTCTTTTTTACCTTGGGAAAAAGTTCCCTTTTTCAGTTAAAAACGTGTTACTATTTTATCGAACCTGGACGGACCAACGGTTGTACCGGTGCTCCAGCAGCATGTCCGGTTCATTCGTCGGTCTAGGTTTTCAAAACTATGCTCTAATGATGTTGGATTTACAATCACACTGATGCGATTTATGTGCGTGGCGCTGACTGTGCTTCCGTTTGGCCAGACCGGTGGACTGTTCGTTGCTGCTCTTTCGGTGATGTTCCCTGTAGTTGGCAACGTTCTGGGTGTTGTGTTTGGTCGTTGCATGGTACTCACTCCGTTCGGAAATACTTGTCCTAGAAATGGTTGTATTTAGACTTATTTTAGTTATAGATACATCTATTTTATCCATTTGTCAGTATTTTTGGATAGAGGAAGTAGGCGGTACTTCAATGAACTACGTGTGGTTGTTTGTGCGGGTTTGATTGCCGGGGGTGGTGCCTTCGTGGTCAAACCTGCAAAATCGCATACGGAGGACATGAATATAGCAAAGGCGTAGCGGAGCGCGCCATTCTGTCTAGTTCATTTTCGTATCATATGGTTACTGTCTTCAGTTTTTTGCTGTTGTAACTTGCTACCTTGCTCTGTTTTATATCTCTGTTTTTGGATCTGTGCATGCTATTTCCTTTCTGGATCACATCCCATGTCTTCTTGAGCAATAGTAATACACTGATGCAATGAGGAGACACACTGCTGACTCTCACTCGTTTGTTCCACCATTGATGTTTGTTCCAAGCTTGACCGGGAGCAGTGGGTTAGGTTGGACCAATGACAGAAGTGGTGAGGCATTCATGTGGAAGTTTATCTCATTACACTGGACTAGAGTCAACGGCTGTTAACATGCTTCTTGATGTCGCCCGTTCCGAAGAAGCAAAGCCAAACAAATTCCTGCCTCCCCTTGGGCATCTAAGACCTGCATGCGTTCTTGTCGAGAGAGGATTGAGTATGTAGATATTTATAGATGTAGAATAGTAAGAAAGAGTCGCAAAAAAAAAAGCGAGGAAAATTATGAGGTGGAAACATTTAAGAACCTAGATGCACATAAGTAATTAGAGGTACTGCTCCGTCAAAACTTTACTACGAGACTTTGGGCATGATAGGTAGGCTGCATCAACCGATCAGGAATGTCACACACACAAGCTATGACTGTCTGGTTATCTGTATGCGCCCACCATATGTTAACATGAGTTGCTCCATTGCTTACAATGACTACTCTAGACGACCGT

**>citrate_synthase_4**

CTCAGGACAACCTTCCGGGAGCGTTAGTGCATTTCATAAAACAATGATGTAAAGGGGGACATCCACGATACAACATATTTGTTGGCCCTCAAATGTCACTTCTATCTCCATGTATATTGGAATTCCAAAGAGCGGTCTTAGGGACTTGTTCCCTGTCCAGCACCATGCTCCAACGCTCAGGTCCAAACCATAAAGCAGGTTACAGTGATACATAGTCCAGATGGTGTTGAGCAGAAAAAAAAACCCACAATATATTACACAAGAAAGAACATGCCATTCTTGTACATAAGTGAACCCCTAAAAAAGCAATGCCCTAGATTACTTAGGTATCATCTTCACACCCCCATGAGACTCCAGGTTGTAGCCTTGAAGATAAATATAGACAAACCCTTCAAGTGACGCCATATATCGGATGATTGATTCGGAGCGGCCTACATCGGAGCAGTTGGGTATCCACTCTTTATCTGCCCTACGGCATAGAGCTGGAATAAAAAGTAGGAGCTCATCATTTCGCATACTTGAACATGCCCCCACAAGCCAATAAACATTCCATTACTCCTGAGACCGTGCTGTGGACCAGCCAAACCATTCAGTGTCGCTACAAAAGAAAGGTAAGGATCTAACAGAGCAGTTCCAACCTGTCCAATAGCCATTCAATCCCCTCAAAGCGATGCACCCAACTGAAACCGCGC

**>malate_dehydrogenase_2_[NADH]**

CACCAGGCTGTCTAGTTTTCCCAGGTAATAGTCGGGGATCCCGAGAAAAACAAGTAGATTATAATCAGGTGTCAAATTGTTTCACTCTTCCAGCAAATGTACTGTCCTGTAAATCATCAGGCAAAAGGGGAGCTACAAGCCTCATTCATACACACTGGGAAAACCAGCGAAAAGGAGCATGCATCGCGATGTGCGGCTGTTCTGGGTAGAGACGAGAGTTTCTAAGGAGCGGAAGGTTTCATTTATTCAAAACATCCAGAGGAGCAGCTGCCCTTCCAATGTGGATTTACGCGAGGCATGAGTAGGCCAGCGCCTTCTCCTCGGACAGCTCCTGGGCGGTCGCATCCATCTTCTTCCTCGAGAACTCATCGATCGGGAGCCCTTGANNAAGACCAGCAGGCACACCGTATGAACCATCAGAGTACACACCCATGGAAACAAATGTTCCCTCGGCGGTTCCCAGAACCCAGTCACGGATGTGATCACAAGCTGAGCTGGCAGCAGAGAGAGCACTGGAGAGCTTCCTCGCTTTGATGATTGCGGCACCACGCTGCTGGACAGTGGCAATGAACTCCCCATTTAGCCATTCATCATCTTGAACAAGTTCACGGACAGGCTTCTCTCCACTGGGAGTCTTCACAGTGGCGTGGTTAACATCAGGGTACTGACTGGACGAGTGATTACCCCAGATAATAGCATTCTTCACGTCAGAAACTTGGACACCAAGTCTCTCAGAAATCTGACCGAGTGCCCTGTTGTGGTCTAGG

**>nitrate_transporter_1.2**

CAGACGGCGTCGGAGAGGAAGCCGCCGAAGAGGCCGAGCAGGAAGGCCGTCCCCATGAAGTTGGTCACCGTCGTGGCCGACTGCGCCGGCGAGTAGTGCATGGACTTCATCAGGTACGTCACCAGGTTACTCGCGTTCGCCAGGAACGCCAGGTTCTCAAGCACCTCCGCAACCAGCACGAAGGAGGCGGCGACCATGCCGCCGTGCCGGCCCTTCACCGCCGGCCGGCTCCTCCAGNNCCGTCCTCCATGGCGCCGCCCGCCATCAAGCCGCGGCGTGCGTGTGTGTGTGAACTCTCAAAGTCTGAAACTCTGAACTGCCGGATGAAGAGAGGAGGCCGTTCAGGAAGGCTCTCAGTCTCAGGGGAGCGCCGTGTGTTGTGTGTGAC

**>nitrate_reductase_1**

GCGGGAGAAGGTCCCCTGCCGCCTCGTCGACAAGAAGGAGCTCTCCCACGACGTCCGCCTCTTCCGCTTCGCGCTGCCGTCCTCCGACCAGGTGCTCGGCCTCCCCGTCGGCAAGCACATCTTCGTGTGCGCCACCATCGACGGCAAGCTCTGCATGCGCGCCTACACGCCGACCAGCATGGTGGACGAGATCGGCCAGTTCGAGCTGCTCGTCAAGGTCTACTTCAAGGACGAGCACCCCAAGTTCCCCAGCGGCGGCCTCATGACGCAGTACCTGGAGTCGCTCCAGCTCGGCTCCTGCATCGACGTCAAGGGCCCGCTGGGGCACGTGGAGTACACCGGCCGCGGCAACTTCGTCATCAACGGCAAGCAGCGGCGGGCGCGCAGGCTGGCCATGATCTGCGGCGGCAGCGGGATCACGCCCATGTACCAGGTGATCCAGGCCGTGCTGCGCGACCAGCCGGAGGACGAGACGGAGATGCACCTGGTGTACGCGAACCGGAGCGAGGACGACATCCTGCTGCGCGACGAGCTGGACCGGTGGGCGGCCGAGTACCCGGAGAGGCTCAAGGTGTGGTACGTGATCGACCAGGTGAAGCGGCCGGAGGACGGGTGGAGGTTCAGCGTCGGGTTCGTGACGGAGGACATCCTGCGGGCGCACGTCCCCGAGGGCGGCGACGACACCCTCGCGCTCGCCTGCGGGCCGCCGCCCATGATCAAGTTCGCCATCTCGCCCAACCTCGAGAAGATGAAGTACGACATGGCCAATTCTTTCATCTCCTTCTGATGAAACATGTTCTCTAGCATGCAGCTGCCTAAGCTATATACCACCGCGTGGACGCATCAATCTGTGGATGTACCCAATGTTTATACGTGTCTGTCCATCGTATTTGTT

**>nitrate_reductase_2**

GGTGAACTGCTTGTCCGCGACGGTGTTCATGAACGGCGTGGAGGTGCTCCGCTTGAGCCCCGGCGCCGCCGCCTCGGCCGTCTCCAGGTGCTTCTGCCGCGCCATCCAGCCGCCGGTCTGGTTGCCGGGCTGCGTGGGGTGCTCGAAGACCAGCCCGATCTCGCCCTTGTGGGGGCGGCACACGTTGATCTTCACCTTGAACCAGCAGTTGTTCATCATGCCCATGAGGTTCCAGATGAGCTTCTCTGGCTGGGTGTTGTGCGTCTGGTCCCATGCGCGCACCGCCACCTCCTTGGCGCCTAGGAGGTCTAGCACCTCGATCTCAACGGACCAGAAGCACCAGCACCAGTACCGGCCGTACTTGTTGGACTTCTCCGGGATGTCCAGCGTGCATAGCATCCAACTCTCACCGCCGTCGAGCGTCACCTCCACTCGCGTGACCTTCTTGCCACCGCCGGAGTATGCGTATCCCTTGATGGTGTAGGCGCGCTGGGTGGTGAAGGCGTTGATGGGGAGGATCTCGTCGTGGCCGGGCGTGGTGATCACCGAGTTGGTGTTGAGCTCGTTGATGATGTACTCGGGGCGGTACCACCACGCCTCCGCGTTGGCCAGCTCGGCGTCCACGTGCGATGGCAGCACCCGGTTGTCCCTGAAGTGGTA

**>ferredoxin_nitrite_reductase**

ACCAGGTCCTCGCACGGCACCGCCTTCTTGTACACCCCCGTGAGGTGCGAGTCGCTGCCGACGCGGCCGCCGACGAAGATGTCCGCCGCCTCGACGATCTTGCCGCTGCTGTTCTTGGTGAGGCAGCCCATGAAGCCGATGTCGGCCACCTGCACCTGCGCGCAGCTGTTGGGGCACCCCGTCCAGTGCATGCGCACCGCCCTGGGCACGGACACGCGCGCCTCCACGTCGCGCGTCACCTGCAGCGCCCGCGCCTTGGTCTCGATGATGGCCTGCCCGCAGAACTGGTTGCCGGTGCACGCCACCAGCCCCTTCATCAGCAGCGACGGGTGCGCCGAGAACTTCTGCAGCAGCGGCTCGGCGAGCAGCGCCTCCACCTTATCGTTCCTGACGTTGGGGAGCACGATGTTCTGCTCCACGGTCAGGCGGAGCTCGCCGGAGCCGTACTCGTCGGCGAGGCGGGCCAGCTCGAACATGTCCGCGGCCTGCAGCCGGCCGACGGGCACGTGCAGGCCGACGAAGGAGAGCCCCTCCTGCTTCTGCGGGTGCACGCCGAGGTAGTCACGCCGCTCCCACTTCTTGTCGATCAGGTCCTCGGCCGCCGCGCGCTCCAGCACGCCGTTCGGCATCCTCTTCTCGATCTCCGACCGGAACGCCTCC

**>glutamine-dependent_asparagine_synthase_1**

AATCACATCCTCAATTGCATCAATGCCGTCCTGAACAGTGAAGGTGAACTCATGGTGCATGGTGCCCAGGTAATTGGCTACCTCCTTTGCAGCCTTCAGATCAGGTGACCCCTCAAGTCCGACACAAAAAGAGTGAAGCTTAGTCCCCCAGCGCTTTGCAGCCTTTGTTCCTGCCAGGTGGCGAACTGTAACGGCTGCAACCAATGATGAGTCAAGGCCACCAGAGAGTAGAACACCGAATGGAACGTCCGTCATAAGCCTCTTGACGACAGCCTTTTCGAAAGCCTTCCTGAGAGCAAGTGGGTCATATGGCACTGAAGGAATGACCTCGGAGAACCAAGGTGGGTTGTACCATCTCTTGAAGCCTCCCTGCTTGCTGGAGTAGAGATGGCCAGGAGGAAAGATCTCAAAGTGCTCACAATCATCATTCAGGCCCTTCATCTCTGATGATATCCACACCGACCCATCAATTCCCCAGCCAATATAGAGGGGTGTGACGCCAATGGCATCACGTGCAGCAATGAAGCTGTTGTCGCGTGTATCGAGCAAGACGAAGGAGAAGACACCATCCAGCATGTCGATGAAGTTCTCCCCATGCTCCTCGTACAGGTGTGCGATGACCTCGCAGTCGCTGCCTGTCCTGAACGTGTGGGAGGAGAGCTGCGCCCGGAGCTGTTCATGGTTGTAGATCTCTCCATTCACTGTGACGACGATGGACTTGTCCTCGTTGTAGAGCGGCTGGTCGCCAGAGGCAGGGTCGATGATGGCGAGGCGCTGGTGGGAGAGGTAGCAGTCGCCAACCTGGTGCATGCCGCTCCAGTCGGGGCCGCGGTGCTTGAGCCTGCGCGAGAGCTCAAGCACGCGCACTCTCTTCCCTTGGGTGTCATCAGCGCAGCCCAGCACCGCCAGTATGCCGCACATTTTCGCTGGATCGACGGCAACAGTATGTAGCTAGAAGATCTCTCTCTGTGCTTCTGAAGGAGGATGGGTGTGCCGTATGAGTTGCACAAGAGCGTCTATTTATAGGCTGCCAAGAGCTGAGCT
GAGCTGAGA

**>aspartate_aminotransferase**

AAGACAATTACATTACACCAAATGAAAAGTTTGAAACAAAGCAAATCGCTCGCAAGTTTGCAATCAAAAAAATGGTGAACTATCTGTTGTACAACAATGGCATGCTTCCTATTTTCCCAATGGACATGCTTTTAATATAATGCTGGTGAAAATGCCTTCAGCGCGCTATTTTTATTGAACCGCGGCAGGGGCCTTGAGCAGAGCCATTGCGTCTTTTATCTTCTCCATCGCAGATTGTAGCGTTGACAGTGCAGCAGCATAGGAGATACGAACACCCTTGTCATCTCCAAATGCATCCCCAGGGACAAGTGCAACCTGTGCTTTCTCTAGCAGGAACATGCAAAGGGTCTCAGAATCCTTGATGGTGCCAAATCCCTCTACCTCGGATCCGTAGTAGGAGCTGAAGTCGATGAACAAATAGAAAGCACCCTGAGGTTCTGATATTTTTACTCCTGGCAGTTCCCTGAAATTTCTTACAAGGTAATCCCGGCGCTCCTGGAATGCTTTGACCATGGTTGAAACTGCTTCACCACCGGCATAGCCAAGGTTCAAGGCTGCAAGCCCTGCCTTCTGTGATATACTACTGGCACCAGATGTGTACTGGCTTTGGATCTTTCCACAGGCTGAGACAAAATGCTTAGGGGCAGCTAAGTATCCAAGTCGCCAACCAGTCATAGCAAAAGCCTTAGAAAACCCATTTACAGTTAATGTTCTGTCATACATTCCAGGTAATGCAGCAAAGCTTGTGTGTTTAGCAGGATGATAGATAATATGCTCNNTCAGATAGAACCAGGAGCCTAGGATACTTCTTGACTATAGCAGCAATCTCCTCAAGCAGCTCCCTAGGATACACAGAGCCTGTTGGATTAGATGGAGAGCATAGAATCAAGAGCCTTGATTTTTCAGTGATCACTGAGGCAAGTGATTCTGGCTTTAGCAGGAAATTGTCTGATATGCTTGTCGGGAGGATCACTGGTGTAGCACCAGCCAACCTAGCCATTTCAGGATAGCTGACCCAAAATGGTGCTGGTATCAAAACCTCATCACCAGGTGAGCAAACAGCAAGAACAGCTTGTGTAATGCACTGTTTAGCTCCATTGCTCACTAGCACCTGATCTGGGCTGTAGGTTAGACCATTCTCCTCCTGAAGCTTGTTGCAGATAGCTTTCCTCAGCTCCATAGTCCCTGCATTAGGTGTGTACCTAGTAGATCCATCCCTGATTGCATTTATCCCAGCCTCGGCGATCACCGCCGGCGTGTCGAAGTCGGGCTCCCCAGCGGCGAGTCCGATGACGGGCACCCCGGCCTGCCGCAGCGCGCTGGCCATGTCGGTGATGGCCATGGTCTTGGACGGCCGCAGCGCGCTCACCCGCGGGCTGATGGACGTGTCCACCGCCTCCGCCCTCACCGCCGCCATCCTGAACTTCCCCGTGGACGCCCTGGAGAAGGAGAGGGAGCCTGCGCCCGGGGAGGGCTTGGTGGGGAGGGTGAAAGACGAGGAGGGGGAGGAGGAGGAAGGGGTGGCGAGGGAGGAGAACGCCATTGGAGCTGTCTGTTTACCTTGCTGGTGCGTGAAGTTGCAGGT

**>glutamate_synthase_1**

GATGGTCACTGTGGGCATCTTGCCATTTCGCATCAAATTGACAGTTTTGTTGCTCAGCCTCCATTGCCGGCATGTTTCCAATGCCAGATATGGGCATATAGCACTGGCTCCATATCCTATCAAACAGGCAAACTGATGGGTGCTGAAACACTGAGCAGTGTCAGCAACAATTGAAGCTGACATGCGGAGGCCATTCTGAATCAAATGCTGGTGGATGGCACCAACAGCTAAAAGTATTGGGACAGCAGGCCGTGTTGGTTCAAGTGCTTCAGAACGATCGGAAAGCACAAGAAGTTGAGATCCACTCCGCACAGCAGCATCAGCTTCTTCACAAAGAGCCTTAATTGCATTCTCAAGGGAACCATCTAGACCTTTACGGATATTGAAGTATGTTGAGAGTACTTTGGGCTTTAATTTTGGGTCCTTCAATAGTGATTCTAGTTCACCTTCATTCAGGACAGGACTTGATAGGGTAACCTGGTCAGCATTTTCAGGGCCAACCTCCAAGATGTTGCCTCGTTTACCAATGTTA

**>glutamate_synthase_2**

TAGTCAGAGAGAAGAGCTACGCGCTAGGTTCCCTGGTGTTCCTGGTGATCTTGTGAATTACTTCCTCTTTGTTGCAGAGGAGGTACGAGCCACATTAGCCCAGTTGGGTTATGAGAAGCTGGATGATATAATTGGGCGGACAGATTTACTTAAGCCAAAGCATATCTCTTTGGTGAAAACGCAGCACATTGATCTTGCATACCTATTAATGAATGCTGGATTACCCAAATGGAGCAGCTCTCAGATAAGGAGCCAGGACGTCCACTCTAATGGCCCTGTTCTTGATGAGACAATCCTTGCAGATCCCGAGGTATCCGATGCAATTGAGAATGAGAAAGAGGTTTCAAAGACATATCCAATTTATAATGTTGATAGGGCTGTTTGCGGCCGTGTAGCGGGTGCCATTGCTAAGAAGTACGGAGATACAGGCTTTGCAGGCCAGCTGAACATTACGTTCACCGGAAGTGCAGGGCAGTCCTTTGGTTGTTTTCTGACACCTGGCATGAATGTTCGCCTAGTAGGAGAGGCCAACGATTATGTGGGAAAGGGTATGGCTGGTGGAGAGCTGGTTGTAGTTCCTGTAGATGATACAGGATTTGTTCCTGAGGATGCTGCTATANNATTTGCAGTTCGGAATTCTCTTGGGCAAGCAGTGGTTGAGGGCACAGGAGATCATTGCTGCGAGTACATGACCGGTGGATGTGTAGTTGTACTCGGCAAAGTTGGAAGAAACGTTGCAGCCGGAATGACCGGTGGCCTAGCCTACATGTTAGATGAAGATGATACTCTAGTCCCGAAGGTAAACAAGGAGATTGTCAAGATGCAGAGAGTGAATGCTCCNNGGCAGCACAAAAGGCGCTAAAATTCTGAGCGAATGGGAGGCATATCTGCCACTCTTCTGGCAGTTGGTGCCACCCAGCGAAGAAGACTCGCCTGAAGCTTGTGCCGAGTTTGAGAGAGTACTTGCCAGGCAAAAAACGGCAGTACAATCTGCTAAGTGATACTAGCCAAAGCCCAATCGCCAAAATGACCAAAGCTAAACGGAGGGAAAGCGTTCTTACTGCGATACTTGAAAGTGATTTTCTCAGACCGCGGTCCTTCTGAAGCACTAACGTCACCAAAGGCTGCAATTTTGTGGAAGCTCTGTCCAAGGCATCTTCTGGAAACGAGCTTGCAGATACCGTCGAGGATTCTTCTGCACGCTCTTGCAGCCTAGGTAACCAACCACCCCTGTAAATTGGTTCGCTGACGCTAGGCTGCAAGGCCCGGCCCCTGTTGAACTGCTGCTTTCTTGTACATGATTCCTGGCTTCGCCTGTGTAATTATATTTTTTGTTCTGCACACATTAGTTCTTATATTTATACCCGTCCTGTGTTAGGAATGCGAGTTGCATGTGCTGTAGTTCAGTACAGAATAAGTAAAGTTTATGATGAAGTAGGACTTCTCCGGTGTCGACAAAAA

**Reference**

Krasileva, K.V., Buffalo, V., Bailey, P., Pearce, S., Ayling, S., Tabbita, F., Soria, M., Wang, S., Akhunov, E., and Uauy, C. (2013). Separating homeologs by phasing in the tetraploid wheat transcriptome. *Genome Biology* 14**,** R66.
