# Supplementary material for: Effects of different nitrogen fertilizers on two wheat cultivars: An integrated approach
Source: Plant Direct. 2018 Oct 22;2(10):e00089. doi: 10.1002/pld3.89 (PMC6508776; doi:10.1002/pld3.89)
Supplement: Supplementary file 2 [file PLD3-2-e00089-s002.pdf]

Manuscript 2018-00161R1

Effects of different nitrogen fertilizers on two wheat cultivars: an integrated approach

Dear Editors,

thanks for your letter of 3rd July.

My coworkers and myself have been involved in revising our manuscript as required, trying to give you complete and satisfactory answers to all questions raised.

Reviewer 1:

Title and abstract have been modified as suggested by the Reviewer. Discussion and results have been reformulated as indicated by the Reviewer. In particular, some parts of the discussion were transferred into the results some section, and a small final part has been added.

Other issues.

1) A certain level of variability between the two years of analysis is conceivable, which could be linked to the different climatic conditions that occurred between the two agricultural years considered. In particular, some spots in the gels analyzed during the second year had higher normalized volume levels than those obtained during the first year of analysis. . Despite these premises, some clarifications are necessary:
   - the three biological replicates analyzed came from three distinct pools, one for the first analysis and two for the second year.
   - The spots considered as differentially expressed had a low level of intra-group variance (i.e. between the two years of analysis considered for each sample). For this reason, all the spots that showed a high level of variability between the two years analyzed were not selected.
   - The differences between the two years considered have had a minor impact on the normalization of the data, as the overall average volume data (optical density) were comparable between the replicates of each sample as well as between different samples.

2) The proteomics results were not validated by qPCR because the identified proteins belonged to metabolic pathways often unrelated to each other. For this reason, we decided to focus on the GS-GOGAT metabolism, believing that such genes could be more influenced by the different forms of nitrogen available. Such an approach has been clarified in the main text as well, as requested.

3) The modulation in the metabolic response highlighted by the proteomic and transcriptomic analyzes show that samples differed more according to the cultivar they belong to and, to a lesser extent, depending on the applied treatment (i.e. type of fertilizer used). If we consider the production data, synthetic fertilizers (Urea, Ammonium Nitrate, Rhizovit) have given the best production performances. In the same way, proteomic data show how the treatments with synthetic fertilizers generically induce an upregulation in the two cultivars considered. By way of example, the data reported in spots 15 and 17 show the most significant differences. The Probable glutathione S-transferase GSTU1 protein, identified in spot 17, is an ideal candidate, as the expression of this protein class is influenced, both in the leaves and in the

root, under conditions of nitrogen deficiency. Regarding the transcriptomic data, the results obtained are strongly influenced by the type of cultivar considered. As for the Creso cultivar, the CS (Creso synthesis) sample has numerous upregulated genes, followed by the CL (Creso leather) sample. The overall trend concerning the Dylan cultivar is different, where a high number of induced genes is reported in the samples treated with protein hydrolysates (DHP1, DHP2) and, to a lesser extent, in the sample treated with leather (DL). From the physiological point of view, these results could be explained by specific characteristics related to the two cultivars, and in particular to the form of preferentially absorbed nitrogen (nitric or ammoniacal). In fact, the fertilizers used, except synthetic products (Ammonium Nitrate and Rhizovit, Supplemental Table 1) do not contain any forms of ammonia nitrogen but contain nitrogen exclusively in the nitric form.

The paragraph above has been introduced in the Discussion section of the revised manuscript.

Reviewer 2:

1. Abstract has been modified according to the Reviewer's indications.
2. Some parts of the text have been deleted (see changes highlighted version of the manuscript), Table 1 of the original version of the manuscript has been included in the new Figure 1, while Table 2 and 6 of the original version have been moved to Supplemental data, as suggested. Figures 5 and 6 have not been merged, since the integration of the two images would lead to some problems of legibility (too small characters).
3. The parameters used for spot selection are those used by default for the analysis carried out in our laboratory (e.g. see Fanucchi F. et al. "Acclimation increases freezing stress response of Arabidopsis thaliana at the proteome level." Biochemistry et Biophysica Acta (BBA) - Proteins and Proteomics1824.6 (2012): 813-825.
4. As now specified in the discussion (line 596), the differences between the two cultivars about the expression of the nitrate transporters could be linked to the form of nitrogen preferentially absorbed by the cultivar (nitric or ammonia form). In the work of Curci et al., 2017 the effect linked to the nitrogen deficiency in the Svevo cultivar was evaluated, using a predominantly molecular approach. In this case, the results show that high-affinity transporters, and in particular NRT2.5, are upregulated in the roots, while no phenomena of up- or down-regulation in the leaves have been highlighted. The Svevo cultivar is a relatively recent cultivar (1996) and has characteristics comparable to the Creso cultivar.
5. Some Authors recently stated that the current understanding of N effects on photosynthetic electron transport rate and partitioning, as well as its impact on photosynthesis under [CO_2]e, is inadequate. (Zhang X.C., X. F. Yu, Y. F. Ma, Effect of nitrogen application and elevated CO_2 on photosynthetic gas exchange and electron transport in wheat leaves. Photosynthetica, vol 51, 593-602, 2013); however according to the data reported in the paper, they concluded that sufficient N improved light energy utilization in wheat flag leaves thus benefiting to photosynthetic assimilation.

   Also Zhou Y-h et al. **(**Effects of nitrogen form on growth, CO_2 assimilation, chlorophyll **f**luorescence, and photosynthetic electron allocation in cucumber and rice plants**.** J Zhejiang Univ Sci B 12:126-134, 2011) found that the N source had little effect on photosynthetic electron allocation in rice plants, except that NH_4^+-grown plants had a higher O_2-independent alternative electron flux than NO_3 -grown plants. NO_3 reduction activity was rarely detected in leaves of NH_4^+-grown cucumber plants but was high in NH_4^+-grown rice plants. These results demonstrate that significant amounts of photosynthetic electron transport were coupled to NO_3 assimilation, an effect more significant in NO_3 -grown plants than in NH_4^+-grown plants.
